# NetCoMi: network construction and comparison for microbiome data in R

**DOI:** 10.1093/bib/bbaa290

**Published:** 2020-12-03

**Authors:** Stefanie Peschel, Christian L Müller, Erika von Mutius, Anne-Laure Boulesteix, Martin Depner

**Affiliations:** Institute for Asthma and Allergy Prevention, Helmholtz Zentrum München, German Research Center for Environmental Health, Munich, Germany; Department of Statistics, LMU München, Munich, Germany; Institute of Computational Biology, Helmholtz Zentrum München, German Research Center for Environmental Health, Munich, Germany; Center for Computational Mathematics, Flatiron Institute, New York, USA; Institute for Asthma and Allergy Prevention, Helmholtz Zentrum München, German Research Center for Environmental Health, Munich, Germany; Dr von Hauner Children’s Hospital, LMU München, Munich, Germany; Comprehensive Pneumology Center Munich (CPC-M), Member of the German Center for Lung Research, Munich, Germany; Institute for Medical Information Processing, Biometry and Epidemiology, LMU München, Munich, Germany; Institute for Asthma and Allergy Prevention, Helmholtz Zentrum München, German Research Center for Environmental Health, Munich, Germany

**Keywords:** compositional data, microbial association estimation, sample similarity network, differential association, network analysis, network comparison

## Abstract

**Motivation:**

Estimating microbial association networks from high-throughput sequencing data is a common exploratory data analysis approach aiming at understanding the complex interplay of microbial communities in their natural habitat. Statistical network estimation workflows comprise several analysis steps, including methods for zero handling, data normalization and computing microbial associations. Since microbial interactions are likely to change between conditions, e.g. between healthy individuals and patients, identifying network differences between groups is often an integral secondary analysis step. Thus far, however, no unifying computational tool is available that facilitates the whole analysis workflow of constructing, analysing and comparing microbial association networks from high-throughput sequencing data.

**Results:**

Here, we introduce NetCoMi (Network Construction and comparison for Microbiome data), an R package that integrates existing methods for each analysis step in a single reproducible computational workflow. The package offers functionality for constructing and analysing single microbial association networks as well as quantifying network differences. This enables insights into whether single taxa, groups of taxa or the overall network structure change between groups. NetCoMi also contains functionality for constructing *differential networks*, thus allowing to assess whether single pairs of taxa are differentially associated between two groups. Furthermore, NetCoMi facilitates the construction and analysis of dissimilarity networks of microbiome samples, enabling a high-level graphical summary of the heterogeneity of an entire microbiome sample collection. We illustrate NetCoMi’s wide applicability using data sets from the GABRIELA study to compare microbial associations in settled dust from children’s rooms between samples from two study centers (Ulm and Munich).

**Availability:**

R scripts used for producing the examples shown in this manuscript are provided as supplementary data. The NetCoMi package, together with a tutorial, is available at https://github.com/stefpeschel/NetCoMi.

**Contact:**

Tel:+49 89 3187 43258; stefanie.peschel@mail.de

**Supplementary information:**

Supplementary data are available at *Briefings in Bioinformatics* online.

## Introduction

The rapid development of high-throughput sequencing techniques [1–4] offers new possibilities for investigating the microbiome across different habitats and provides the opportunity to discover relationships between the composition of microbial communities and their environment. Sequencing technologies include shotgun metagenomic sequencing, where whole genomes of microbes in the sample are sequenced, and targeted amplicon sequencing, where microbial abundances are derived from sequencing a specific marker gene (e.g. 16S rRNA, 18S rRNA or ITS) [5]. Following McLaren *et al.* [6], we refer to marker-gene and metagenomic sequencing techniques as *MGS* techniques.

MGS techniques allow the quantification of taxon abundances in the sample in form of operational taxonomic units (OTUs) [7, 8], amplicon sequencing variants (ASVs) [9], or metagenomic OTUs (mOTUs) [10]. However, due to limitations in the sequencing process, MGS measurements often provide only relative or compositional information with each component expressing the relative frequencies of a taxon in the sample [11]. Furthermore, the observed MGS measurements represent only a sample of the true microbial composition present in the biological material [12]. Accordingly, it is likely that not all taxa occurring in a sample are measured due to technical limitations in library preparation and the sequencing process [12]. The observed number of reads is thus only a noisy measurement reflecting the probability of the corresponding organisms to be present [11]. Moreover, MGS data collections comprise a high amount of zero counts, contain samples with varying sequencing depths (sum of counts per sample), and are usually high-dimensional, i.e. the number of taxa $p$ is much larger than the sample size $n$.

A common exploratory analysis approach for microbiome survey data [13–17] is the estimation of microbe–microbe association networks [18–21], allowing for high-level insights into the global structure of microbial communities. Although a wide range of general-purpose tools is available for visualizing and analysing networks [22, 23], including the R packages igraph [24], statnet [25] and network [26], the special nature of current MGS data requires careful statistical treatment. For instance, statistical network analysis methods ignoring the compositional structure of the data may lead to spurious results (also known as *compositional effects*) [11]. Existing *compositionally aware* approaches for measuring and estimating microbial associations include correlation estimators, such as SparCC [27], partial correlation estimators [28, 29] and proportionality [30].

Although the analysis of a single microbial association network can provide insights into the general organizational structure of a microbial community, researchers are often more interested in how microbial associations *change* across different conditions. For instance, it is often desired to find relationships between microbial compositions, their inherent connectivity and an underlying phenotype, e.g. the health status of patients. This task thus requires the quantitative comparison of networks across conditions.

Current approaches for comparing networks between two conditions can be divided into two types: (i) *differential association analysis* focusing on differences in the strength of single associations, and (ii) *differential network analysis*, analysing differences between network metrics and network structure between two conditions [31]. Differential associations can be used as the basis for constructing differential networks, comprising only differentially associated node sets. Existing tools for network comparison either require pre-computed networks [32] or are tailored for specific biological purposes, including protein–protein interactions [33, 34] or gene functions [35].

**Figure 1:**
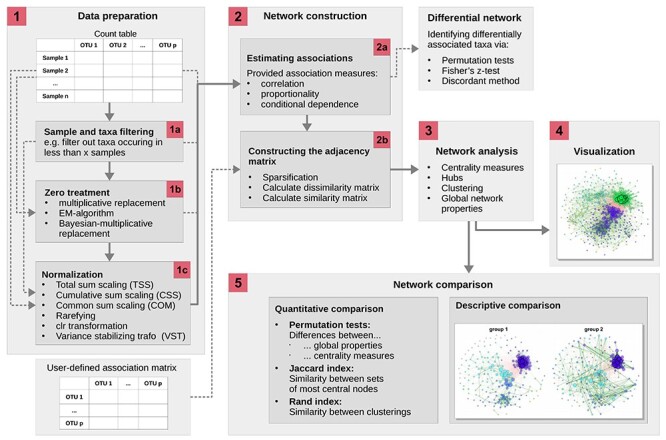
The proposed workflow for constructing, analysing and comparing microbial association networks, implemented in the R package NetCoMi. The main framework (displayed as continuous lines) requires a $n \times p$ read count matrix as input. The data preparation step includes sample and taxa filtering, zero replacement and normalization (step 1). Associations are calculated and stored in an adjacency matrix (step 2). Alternatively, an association matrix is accepted as input, from which the adjacency matrix is determined. A more detailed chart describing step 2 is given in Figure 2. In step 3, network metrics are calculated, which can be visualized in the network plot (step 4). If two networks are constructed (by passing a binary group vector, two count matrices or two user-defined association matrices to the function), their properties can be compared (step 5). Besides the main workflow, a differential network can be constructed from the association matrix.

In this paper, we introduce NetCoMi (**Net**work **Co**nstruction and comparison for **Mi**crobiome data), a comprehensive R package, that specifically focuses on microbiome data analysis. NetCoMi integrates previously disjoint microbial network inference and analysis tasks into a single coherent computational workflow and allows the user to construct, analyse and compare microbial association networks in a fast and reproducible manner. The complete NetCoMi framework is shown in Figure 1. NetCoMi provides a wide range of existing methods for data normalization, zero handling, edge filtering, and a selection of association measures, which can be combined in a modular fashion to generate the microbial networks. For network analysis, several local and global network properties are provided, which can be visualized in network plots to enable a descriptive comparison. Quantitative comparison between two different networks is available via the integration of appropriate statistical tests. Furthermore, our package enables (i) the generation of differential microbial networks and (ii) the construction of *sample similarity networks* (using, e.g. the Bray-Curtis measure), which can serve as a high-level visual summary of the heterogeneity of the microbiome sample collection.

## Network construction and characterization

### Data filtering, normalization, and zero handling

The process of constructing microbial association networks starts with a matrix containing non-negative read counts originating from a sequencing process. Although NetCoMi can handle any kind of count abundance data as input, our main focus here is on compositional read count data coming from MGS experiments. The total read counts $\omega ^{(k)} = [\omega _1^{(k)},..., \omega _p^{(k)}]$ of a sample $k$ with $p$ taxa are a composition summing up to a constant $m^{(k)} = \sum _{i=1}^{p} \omega _i^{(k)}$, the sequencing depth. The sequencing depth differs from sample to sample and is predefined by technical factors leading to sparse data with many zeros. Thus, preprocessing steps (step 1 in Figure 1) are recommended, or even mandatory depending on the association measure (Supplementary Table S1).

To simplify the graphical interpretation of an association network and the computational processes, it is reasonable to filter out a certain set of taxa as first data preparation step (step 1a in Figure 1). See Table S2 for the options available in NetCoMi.

The excess number of zeros in the data is a major challenge for analysing microbiome data because parametric as well as non-parametric models may become invalid for data with a large amount of zeros [39]. Moreover, many compositionally aware measures are based on so-called log-ratios. Log-ratios have been proposed by Aitchison [45] as the basis for statistical analyses of compositional data as they are independent of the total sum of counts $m$. More precisely, for two variables $i$ and $j$ the log-ratio of relative abundances $log(\frac{x_i}{x_j}) = log(\frac{\omega _i/m}{\omega _j/m})$ is equal to the log-ratio of the absolute abundances $log(\frac{\omega _i}{\omega _j})$ [28]. However, log-ratios cannot be computed if the count matrix contains any zeros, making zero handling necessary (step 1b in Figure 1). Several zero replacement strategies have been proposed [40–42, 44, 46]. Table 1 gives an overview of the different types of zeros that have been suggested as well as existing approaches for their treatment.

**Table 1:** Different zero types defined for sequencing data with appropriate approaches for their treatment (suggested by Martín-Fernández *et al.* [36]), which are available in NetCoMi. The three approaches are implemented in R via the zCompositions package [37]. Shown are for each treatment method a few key facts as well as the argument for applying it in NetCoMi.

Type	**Essential/structural zeros**	**Rounded zeros**	**Count zeros**
Description	“... component which is truly zero, not something recorded as zero simply because the experimental design or the measuring instrument has not been sufficiently sensitive to detect a trace of the part.” [38]	Assumed to be present in the sample but their proportion is too low to be detected [36].	Sequencing data are seen as categorical. For a zero count it is assumed that, in this category, no event occurred in this experimental run but could occur in another run (e.g. with a different sequencing depth) [36].
Treatment methods	$\bullet $ No general approach available so far [39] $\bullet $ The “structural zero issue is by far the most complicated problem”[39] $\Rightarrow $ no approach included in NetCoMi	**Multiplicative imputation** [40]: $\bullet $ Non-parametric $\bullet $ Small value $\delta $ imputed for all zeros $\bullet $ Non-zero values adjusted regarding unit-sum constraint to preserve their covariance structure $\bullet $NetCoMi arg.: zeroMethod = “multRepl”**Modified EM $alr$-algorithm [41–43]:**$\bullet $ Parametric $\bullet $ Covariance structure is taken into account $\bullet $ Zeros imputed via EM-algorithm for $alr$ transformed data $\bullet $NetCoMi arg.: zeroMethod = “alrEM”	**Bayesian-multiplicative treatment** [44]: $\bullet $ Parametric $\bullet $ Observed counts $\boldsymbol{c}=(c_1,..., c_p)$ assumed to be categorial $\bullet $ Random vector $C$ multinomial distributed with parameters $(n, \pi _1,..., \pi _p)$$\bullet $$\boldsymbol{\pi }$ corresponds to observed microbial proportions $\rightarrow $ estimated via posterior distribution $\bullet $NetCoMi arg.: zeroMethod = “bayesMult”

Normalization techniques are required to make read counts comparable across different samples [47, 48] (step 1c in Figure 1). The normalization approaches included in NetCoMi are summarized in Table 2. A description of these methods is available in [47, 48]. Note that forcing the read counts of each sample to a unique sum (as done with total sum scaling) does not change the compositional structure. Rather, proportions are always compositional, even if the original data are not [11]. Aitchison [45] suggested using the centered log-ratio (clr) transformation to move compositional data from the simplex to real space. Badri *et al.* [47] have shown that variance-stabilizing transformations (VSTs) [49] as well as the clr-transformation produce very similar Pearson correlation estimates, which are more consistent across different sample sizes than TSS, CSS and COM methods.

**Table 2:** Data normalization techniques implemented in NetCoMi. More detailed descriptions of these methods can be found in Badri et al. [47] and McMurdie and Holmes [48]. The corresponding NetCoMi argument is normMethod with the available options: “none”, “TSS”, “CSS”, “COM”, “rarefy”, “clr” and “VST”.

**Method**	**Approach**	**Comments**
Total sum scaling (TSS)	Traditional approach for building fractions. Counts are divided by the total sum of counts in the corresponding sample.	Strongly influenced by highly abundant taxa [47].
Cumulative sum scaling (CSS) [50]	Within each sample, the counts are summed up to a predefined quantile. The counts are then divided by this sum.	Aims at avoiding the influence of highly abundant taxa [47].
Common sum scaling (COM) [48]	Counts are scaled according to the minimum sum of counts over all samples so that all samples have equal library size (that of the sample with minimum overall sum).	Originally suggested as alternative to rarefying as common normalization method [48].
Rarefying [51]	Subsampling from the data to obtain samples with equal library size (also called rarefaction level) [48].	
Centered log-ratio (clr) transformation [45]	For a composition $x = (x_1,..., x_p)$ the clr transformation is defined as $clr(x) = log(\frac{x_1}{g(x)},..., \frac{x_p}{g(x)}),$ where $g(x) = \left (\prod _{k=1}^{p} x_k\right )^{\frac{1}{p}}$ is the geometric mean.	Aims to avoid compositional effects.
Variance stabilizing transformation (VST) [49]	The dispersion-mean relation is fitted based on the count matrix. The data are then transformed to receive a matrix that is approximately homoscedastic [49].	Aims at eliminating the dependence of the variance on the mean. VST and the clr transformation produce very similar Pearson correlations [47].

### Measuring associations between taxa

Association estimation is the next step in our workflow (step 2a in Figure 1) to obtain statistical relations between the taxa. Common association measures include correlation, proportionality and conditional dependence. For all three types of association, compositionally aware approaches have been proposed, which are summarized in Table 3. Further information on these measures is available in Supplementary material 1. To ensure wide applicability of NetCoMi, the package comprises also traditional association measures, which are not suitable for application on read count data in their original form.

**Table 3:** Overview of compositionally aware association measures that are available for network construction in NetCoMi. The corresponding argument is named measure. The measures are grouped by the three types of association: correlation, proportionality and conditional dependence. For each measure, the assumptions stated in the corresponding publication are listed, together with a short summary of the approach.

**Measure**		
** Data transformation** ** R implementation**	**Assumptions**	**Approach**
Correlation		
SparCC [27] $\bullet $ Log-ratios $\bullet $R code based on package r-sparcc [60]	$\bullet $ Large number of taxa$\bullet $ Sparse underlying network (taxa sparsely correlated)	$\bullet $ Idea: estimating Pearson correlations from log-ratio variances via an approximation based on the assumption that taxa are uncorrelated on average $\bullet $ Zero handling: Bayesian approach (observations replaced by random samples) $\bullet $ Nested iterations to reinforce sparsity assumption and account for uncertainty due to random sampling
CCLasso [53] $\bullet $ Log-ratios $\bullet $R code on GitHub [61]	$\bullet $ Large number of taxa $\bullet $ Sparse underlying network	$\bullet $ Idea: inferring correlations from observed counts via latent variable model $\bullet $ Approach: minimizing a loss function (considers errors resulting from estimating the covariance matrix from observed data instead of true abundances) plus a penalty to incorporate the sparsity assumption
CCREPE [18] $\bullet $ Relative abundances $\bullet $R package ccrepe [62]	**–**	$\bullet $ Idea: testing for statistical significance of the estimated correlations, whereby spurious correlations caused by compositional effects are considered $\bullet $ Approach: permuting and re-normalizing the data to assess correlations due to compositionality alone
Proportionality		
$\rho $ [54] $\bullet $ clr transformation $\bullet $R package propr [30]	**–**	$ \rho(\textrm{log}(x), \textrm{log}(y)) = 1 - \frac{var(\textrm{log}(x/y))} {var(\textrm{log}(x))} + var(\textrm{log}(y)) $
Conditional dependence		
SPIEC-EASI [63] $\bullet $ Log-ratios $\bullet $R package SpiecEasi [63]	$\bullet $ Large number of taxa $\bullet $ Sparse underlying network	$\bullet $ Idea: covariance matrix for clr-transformed data is approximately equal to the “true” covariance matrix $\Sigma $ for $p\gg 0$$\bullet $ Zero entries in the inverse covariance matrix $\Sigma ^{-1}$ correspond to conditional independent taxa $\bullet $ Approach: a graphical model is used to infer the conditional dependence structure between each two taxa $\bullet $ Two methods for graphical model inference: sparse inverse covariance selection and neighborhood selection
gCoda [56] $\bullet $ Relative abundances $\bullet $R code on GitHub [64]	$\bullet $ True absolute abundances $y$ follow a multivariate normal distribution $\bullet $ Sparse underlying network	$\bullet $ $log(y) \sim N(\mu , \Sigma )$ with $\Omega = \Sigma ^{-1}$ representing conditional dependence relationships between taxa $\bullet $$\Omega $ is determined by minimizing the negative log-Likelihood for $(\mu , \Omega )$ plus $l_1$ penalty (to satisfy sparsity assumption) $\bullet $ Optimization problem solved via Majorization- Minimization algorithm
SPRING [55] $\bullet $*mclr* transformation$^1$ $\bullet $ R package SPRING [65]	$\bullet $ Sparse underlying network	$\bullet $ $\Sigma $ is estimated using a semi-parametric rank based approach relying on a truncated Gaussian copula model [66], which can deal with zeros in the data $\bullet $ Conditional independence relationships are inferred from $\Sigma $ using neighborhood selection [58] $\bullet $ Graphical model selection via stability-based approach

$^1\; clr$ transformation where only non-zero elements are included in the geometric mean [55].

#### Correlations

Compositionality implies that if the absolute abundance of a single taxon in the sample increases, the perceived relative abundance of all other taxa decreases. Applying traditional correlation measures such as Pearson’s correlation coefficient to compositions can lead to spurious negative correlations, which do not reflect underlying biological relationships. It has been shown that the resulting bias is stronger, the lower the diversity of the data [27].

A possible approach to reduce compositional effects is to normalize or transform the data in a compositionally aware manner and apply standard correlation coefficients to the transformed data. A common method is the clr transformation (Table 2), which is a variation of the aforementioned log-ratio approach. Traditional correlation measures provided in NetCoMi are the Pearson correlation, Spearman’s rank correlation and the biweight midcorrelation (bicor). Bicor as part of the R package WGCNA [52] is more robust to outliers than Pearson’s correlation because it is based on the median instead of the mean of observations.

Popular compositionally aware correlation estimators include SparCC (Sparse Correlations for Compositional data) [27], CCLasso (Correlation inference for Compositional data through Lasso) [53] and CCREPE (Compositionality Corrected by REnormalization and PErmutation), also called ReBoot method [18]. SparCC is one of the first approaches developed for inferring correlations for compositional data and has become a widely used method [53]. However, SparCC has some limitations, namely that the estimated correlation matrix is not necessarily positive definite and may have values outside [-1,1] [53]. Furthermore, the basic algorithm is repeated iteratively to reinforce the sparsity assumption and to account for uncertainties due to random sampling, leading to high computational complexity. CCLasso is also based on log-ratios but aims to avoid the disadvantages of SparCC by using a latent variable model to infer a positive definite correlation matrix directly [53]. The CCREPE approach operates directly on relative read counts, which are permuted and renormalized in order to detect correlations induced by compositionality alone.

#### Proportionality

Lovell *et al.* [54] argue that correlations cannot be inferred from the relative abundances in a compositionally aware manner without any assumptions and propose proportionality as an alternative association measure for compositional data. If two relative abundances are proportional, then their corresponding absolute abundances are proportional as well: $\frac{\omega _i}{m} \propto \frac{\omega _j}{m} \Rightarrow \omega _i \propto \omega _j$. Thus, proportionality is identical for the observed (relative) read counts and the true unobserved counts. Lovell *et al.* [54] suggest proportionality measures based on the log-ratio variance ${var}({log}\frac{x_i}{x_j})$, which is zero when $\omega _i$ and $\omega _j$ are perfectly proportional. This variance, however, lacks a scale that would make the strength of association comparable. For this reason, the proposed proportionality measures $\phi $ and $\rho $ [30, 54] are modifications of the log-ratio variance based on clr transformed data that come with a scale. Due to its analogy to correlations, we included $\rho $ in the NetCoMi package (Table 3 for the formula). $\rho $ is a symmetric measure with values in [-1,1], where 1 corresponds to perfect proportionality.

#### Conditional dependence

Conditional dependence expresses the relation between two variables conditioned on all other variables in the data set [28]. Hence, this is a measure of direct relations between each two taxa, whereas (marginal) correlations cannot differentiate between direct and indirect dependencies. Three estimators of conditional dependence are included in NetCoMi: SPRING (Semi-Parametric Rank-based approach for INference in Graphical model) [55], SPIEC-EASI (Sparse Inverse Covariance Estimation for Ecological Association Inference) [28] and gCoda [56].

All three approaches use graphical models to infer the conditional independence structure from the data. For multivariate Gaussian data, the graph structure can be inferred from the non-zero elements of the inverse covariance matrix $\Omega =\Sigma ^{-1}$ [57], where each entry is related to scaled negative partial correlation. Loh and Wainwright [57] relaxed the Gaussianity assumption and established relationships between the inverse covariance matrix and the edges of a graph for discrete data. All three approaches for estimating a conditional dependence graph presume that the assumptions of the data generation process are fulfilled so that the graph structure can be reliably inferred from the count matrix. We also consider the assumptions on the data generation process as satisfied and use *conditional dependence* and *partial correlation* equivalently in the following.

gCoda uses a Majorization-Minimization algorithm to infer $\Omega $ from the data based on maximizing a penalized likelihood. In SPIEC-EASI, two approaches are provided to obtain $\Omega $ from the observed count data: neighborhood selection [58] and sparse inverse covariance selection (also known as “graphical lasso”) [59]. SPRING uses a semi-parametric rank-based correlation estimator which can account for excess zeros in the data and applies neighborhood selection to infer conditional dependencies. All three measures assume a sparsely connected underlying network.

### Constructing the adjacency matrix

**Figure 2:**
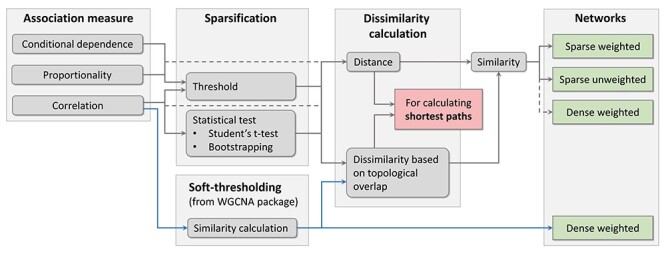
Approaches for network construction that are available in NetCoMi, depending on the association measure. For correlations, in addition to a threshold and statistical testing, the soft-thresholding approach from WGCNA package [52] is implemented (marked by blue arrows). Dissimilarity based on topological overlap (also adopted from WGCNA package) is available as a further dissimilarity transformation approach in addition to metric distances and thus used for all network properties based on shortest paths. Whether a network measure is based on similarity or dissimilarity is stated in Table 4. Network construction without a sparsification step leads to dense networks where all nodes are connected.

Using one of the aforementioned association measures, an association matrix with entries $r_{ij}$ expressing the relation between pairs of taxa $i$ and $j$ is computed. The next step is sparsification and transformation into distances and similarities (step 2b in Figure 1) resulting in an adjacency matrix $A^{p\times p}$ with entries $a_{ij}$ as numerical representation of the microbial network, where nodes (or vertices) represent the taxa. The different options available in NetCoMi are illustrated in Figure 2 (Supplementary Figure S1 for a more detailed version of this chart).

Since the estimated associations are generally different from zero, using them directly as adjacency matrix results in a *dense network*, where all nodes are connected to each other and consequently only weighted network measures are meaningful. Instead, the association matrix is usually sparsified to select edges of interest.

One possible sparsification strategy consists of defining a cut-off value (or threshold) so that only taxa with an absolute association value above this threshold are connected [27, 67]. This filtering method is available for all types of association. The conditional independence measures SPRING, SPIEC-EASI and gCoda already include a model selection approach, making the filter step unnecessary.

For correlations, statistical tests are available as alternative sparsification method, allowing only significant associations to be included in the network. Student’s *t*-test [68] and a bootstrap approach [27, 67] are implemented for identifying correlations significantly different from zero. The resulting *P*-values need to be adjusted to account for multiple testing. NetCoMi includes all adjustment methods available in the R function p.adjust() (stats [69]) and, in addition, two methods for multiple testing under dependence having higher power than the common Benjamini-Yekutieli method [70]: (i) fdrtool() for controlling the local false discovery rate [71, 72], and (ii) the adaptive Benjamini-Hochberg method [73] where the proportion of true null hypotheses is estimated using convex decreasing density estimation as proposed by Langaas et al. [74].

*P*-values arising from bootstrapping might be problematic because they can be exactly zero if none of the bootstrap-correlations is more extreme than the observed one. Since *P*-values of exactly zero cannot be corrected for multiple testing, NetCoMi’s bootstrap p-values are corrected by adding pseudo counts:

$$\begin{align*}& p = \frac{b+1}{m+1}, \end{align*}$$

where $b$ is the number of generated samples with a test statistic at least as extreme as the observed one, and $m$ is the number of repetitions [75].

As illustrated in Figure S1, the sparsified associations $r_{ij}^*$ are then transformed into dissimilarities/distances $d_{ij}$, which are needed for computing network measures based on shortest paths (Section 2.4). Following van Dongen and Enright [76], we included different distance metrics depending on how negative associations should be handled (Figure S1). Available are the options: (i) “unsigned”: $d_{ij}=\sqrt{1-{r_{ij}^*}^2}$, leading to a low distance between strongly associated taxa (positively as well as negatively), (ii) “signed”: $d_{ij}=\sqrt{0.5\; (1-r_{ij}^*)}$, where the distance is highest for strongly negative associated taxa, and (iii) “signedPos”: “signed” distance with setting negative associations to zero. A dissimilarity measure based on the topological overlap matrix (TOM) [77] is also available in NetCoMi.

The adjacency matrix contains similarities of the form $s_{ij} = 1-d_{ij}$. These similarity values are used for network plot and network metrics based on connection strength (Section 2.4). NetCoMi also offers two options for constructing an unweighted network via generating a binary adjacency matrix from the sparse association matrix where the user can decide whether or not negative associations should be included in the unweighted network (Figure S1).

Furthermore, we included adjacency matrix constructions via the *soft-thresholding* approach from the WGCNA package [46, 52], which is only available for correlations (see blue path in Figure 2). Rather than using *hard thresholding* (e.g. via cutoff value or statistical tests), Zhang and Horvath [46] suggested raising the estimated correlations to the power of a predefined value greater than 1 to determine the adjacencies (Supplementary material 2.2 for more details). Small correlation values are thus pushed toward zero becoming less important in the network. The resulting similarities are used as edge weights for network plotting and network metrics based on connection strength. Note that this approach leads to a fully connected network. Following Zhang and Horvath [46], only TOM dissimilarities are available in NetCoMi for computing shortest paths and clusters when soft-thresholding is used.

### Network analysis

We analyse a constructed network by calculating network summary metrics (step 3 in Figure 1), which are amenable to group comparisons. Alternatively, NetCoMi offers the possibility to analyse and visualize single networks.

Several network statistics require shortest path calculations. A *path* between two vertices $v_0$ and $v_k$ is a sequence of edges connecting these vertices such that $v_0$ and $v_k$ are each at one end of the sequence and no vertices are repeated [78]. The *length* of a path is the sum of edge weights, where the weight of an edge is a real non-negative number associated with this edge [78]. The *shortest path* between two nodes is the path with minimum length. In NetCoMi, edge weights are defined in two ways: (i) for properties based on shortest paths, dissimilarity values are used implying that the path length between two taxa is shorter the higher the association between the taxa is. (ii) For properties based on connection strength, the corresponding similarities are used as edge weights (Section 2.3 and Figure S1 for details on distance and similarity calculation).

Network centrality measures offer insights into the role of individual taxa within the microbial community. We consider degree, betweenness, closeness and eigenvector centrality (Table 4). Using these measures, so-called *hubs* (or keystone taxa) can be determined. This analysis step is of high interest to researchers since hub nodes may correspond to taxa with a particularly important role in the microbial community. What is deemed “important”, depends on the scientific context. A selection of definitions for hub nodes is given in Table 4.

**Table 4:** Local and global network properties implemented in NetCoMi. The table shows for each measure a short explanation and whether it is based on connection strength (similarity) or dissimilarity/distance (e.g. two taxa with a high correlation have a high connection strength, but their distance is low).

**Measure**	**Definition**	**Based on**
Shortest path	Sequence of edges connecting two nodes with minimum sum of edge weights [78].	Dissimilarity
**Local measures (refer to single nodes)**	
Degree centrality	Number of adjacent nodes [79]. Options: weighted/unweighted.	Similarity
Betweenness centrality	Fraction of times a node lies on the shortest path between all other nodes [79]. $\Rightarrow $ A central node has the ability to connect sub-networks [67].	Dissimilarity
Closeness centrality	Reciprocal of the sum of shortest paths between this node and all other nodes [79]. $\Rightarrow $ The node with highest closeness centrality has the minimum shortest path to all other nodes.	Dissimilarity
Eigenvector centrality	Calculated via eigenvalue decomposition: $Ac=\lambda c$, where $\lambda $ denotes the eigenvalues and $c$ the eigenvectors of the adjacency matrix $A$. Eigenvector centrality is then defined as the $i$-th entry of the eigenvector belonging to the largest eigenvalue [80, 81]. $\Rightarrow $ A node is central if it is connected to other nodes having themselves a central position in the network.	Similarity
Normalized centralities$^1$	For each centrality measure, a normalized version leading to values in [0,1] is implemented in NetCoMi. We use the following definitions of normalized centrality for a vertex $v_i$: Degree: $C_{\textrm{Deg}}^*(v_i) = \frac{1}{n-1} C_{\textrm{Deg}}(v_i)$ [79], Betweenness centrality: $C_{\textrm{Betw}}^*(v_i) = \frac{2}{n^2-3n+2}C_{\textrm{Betw}}(v_i)$ [79], Closeness centrality: $C_{\textrm{Close}}^*(v_i) = \frac{1}{n-1} C_{\textrm{Close}}(v_i)$ [79], Eigenvector centrality: $C_{\textrm{Eigen}}^*(v_i) = \frac{C_{\textrm{Eigen}}(v_i)}{\textrm{max}(C_{\textrm{Eigen}}(v))}$ [82], where $n$ is the number of nodes in the network, respectively.	
Hubs	Particularly important nodes, which are most central regarding: 1) highest degree centrality [83], 2) highest degree, betweenness and closeness centrality at the same time [84], 3) or highest eigenvector centrality [24]. For each centrality measure, the *most central nodes* are those with a centrality value either above a certain quantile of the fitted log-normal distribution [84] or above a certain empirical quantile.	Depends on the centrality measure.
**Global network metrics (refer to the whole network)**	
Average path length	Arithmetic mean of all shortest paths between vertices in a network [83].	Dissimilarity
Global clustering coefficient	Proportion of triangles with respect to the total number of connected triples$^2$ [83]. $\Rightarrow $ Expresses how likely the nodes are to form clusters [83]. For weighted networks, the definition according to Barrat et al. [85] is used in NetCoMi.	Similarity
Modularity	Expresses how well the network is divided into communities (many edges within the identified clusters and only a few between them) [86].	Depends on the clustering algorithm.
Edge / vertex connectivity	Minimum number of edges, or vertices (nodes) that need to be removed to disconnect the network, respectively [87]. Not meaningful for a fully connected network.	Presence/ absence of an edge
Density	Ratio of the actual number of edges in the network and the possible number of edges [87]. Not meaningful for a fully connected network.	Presence/ absence of an edge
**Clustering**	
Hierarchical clustering	Hierarchical clustering using the R function hclust() from stats package [69]. Different methods are provided (e.g. single, complete, and average linkage, Ward’s method). The cutree() function (stats) is used for cutting the resulting tree.	Dissimilarity
Modularity clustering [88]	The modularity measure is maximized over all possible clusterings.	Similarity
Fast greedy modularity optimization [86]	Modularity clustering via a fast greedy algorithm, which is suitable for very large networks.	Similarity
Clustering based on edge betweenness [89]	Idea: Edges with high edge betweenness (number of shortest paths leading through an edge) tend to divide the network into clusters. Approach: Hierarchical clustering, where edges with high edge betweenness are removed gradually from the network.	Dissimilarity

$^1$ Not to be confused with “centralization” [79], which is a global network measure expressing the degree to which the centrality of the most central node exceeds the centrality of all other nodes in the network. $^2$ A connected triple is a group of three nodes with two or three edges. A triangle is a triple where all three nodes are connected.

Clustering methods are appropriate to identify functional groups within a microbial community. A *cluster* (or module) is a group of nodes that are highly connected to one another but have a small number of connections to nodes outside their module [67]. The user of NetCoMi can choose between one of the clustering methods provided by the igraph package [24] and hierarchical clustering (R package hclust() from stats package). Both similarities and dissimilarities can be used for clustering (Table 4).

Global network properties are defined for the whole network and offer an insight into the overall network structure. Typical measures are the average path length, edge and vertex connectivity, modularity, and the clustering coefficient (Table 4).

### Sample similarity networks

Using dissimilarity between samples rather than association measures among taxa leads to networks where nodes represent subjects or samples rather than taxa. Analogous to microbiome ordination plots, these *sample networks* express how similar microbial compositions between subjects are and thus provide insights into the global heterogeneity of the microbiome sample collection.

The following dissimilarity measures are available in NetCoMi: Euclidean distance, Bray-Curtis dissimilarity [90], Kullback-Leibler divergence (KLD) [91], Jeffrey’s divergence [92], Jensen-Shannon divergence [93], compositional KLD [94, 95], and Aitchison distance [96]. Details are available in Table S3. Only Aitchison’s distance and the compositional KLD are suitable for application on MGS data whereas the others may induce compositional effects when applied to raw count data without appropriate transformation (Table 2).

The workflow for constructing, analysing and comparing sample similarity networks is described in Supplementary material 2.5. For these networks, the same network properties as for microbial networks are available in NetCoMi (Table 4). While the estimated dissimilarity values $d_{kl}$ between two samples $k$ and $l$ are used for network properties based on shortest paths, the corresponding similarities, calculated by

$$\begin{align*} &s_{kl} = 1 - d_{kl},\end{align*}$$

are used for properties based on connection strength and edge weights in the network plot. Accordingly, highly connected nodes are subjects with a microbial composition similar to many other subjects. Furthermore, as in usual cluster analysis, clusters represent subjects with similar bacterial composition but with the advantage, that the solution is visualized in the network plot.

## Network comparison

NetCoMi’s network comparison module (step 5 in Figure 1) focuses on investigating the following questions in a quantitative fashion: (i) Is the overall network structure different between two groups? (ii) Are hub taxa different between the two microbial communities? (iii) Do the microorganisms build different “functional” groups? (iv) Are single pairs of taxa differentially associated among the groups? To meet these objectives, NetCoMi offers several network property comparison modes as well as the estimated associations itself between two groups. These approaches are commonly used in other fields of application and have been adapted to the microbiome context. Each method includes statistical tests for significance.

To perform network comparison, the count matrix is split into two groups according to the user-defined group indicator vector. All steps of network construction and analysis described in (Section 2) are performed for both subsets separately. The estimated associations, as well as network characteristics, are then compared using the methods described below.

### Differential network analysis

We next detail NetCoMi’s differential network analysis capabilities. All approaches are applicable for microbial association networks and sample similarity networks, respectively.

#### Permutation tests

NetCoMi uses permutation tests to assess for each centrality measure and taxon whether the calculated centrality value is significantly different between the two groups. The null hypothesis of these tests is defined as $H_0: c_1^{(i)}-c_2^{(i)}=0$, where $c_1^{(i)}$ and $c_2^{(i)}$ denote the centrality value of taxon $i$ in group 1 and 2, respectively. A standard non-parametric permutation procedure [97] is used to generate a sampling distribution of the differences under the null hypothesis (Supplementary material 3.1). The same approach is used to test for significant group differences in the global network characteristics listed in Table 4.

#### Similarity of most central nodes

A set of *most central nodes* can be defined in two ways: In the first approach, under the assumption that the centrality values are log-normal distributed [84], the set of most central nodes contains nodes with a centrality value greater than a certain quantile of the fitted log-normal distribution. In the second approach, the empirical quantile can be used directly without any distributional assumption. Both approaches are included in NetCoMi, where the quantile can be freely chosen in each case.

The Jaccard index [98] (Supplementary material 3.2) can then be used for assessing how different the two sets of most central nodes (regarding a certain centrality measure) are between the groups. This index ranges from zero to one, where a value of one corresponds to two equal sets and zero means that the sets have no members in common. Following Real and Vargas [99], we included an approach to test whether the observed value of Jaccard’s index is significantly different from that expected at random (Supplementary material 3.2). Note that this approach cannot make a statement on whether the two sets of most central nodes are significantly different.

#### Similarity of clustering solutions

NetCoMi offers several network partitioning and clustering algorithms (Table 4). One way to assess the agreement of two partitions is via the Rand index [100] (Supplementary material 3.3). Like Jaccard’s index, the Rand index ranges from 0 to 1, where 1 indicates that the clusters are exactly equal in both groups. The original Rand index is dependent on the number of clusters making it difficult to interpret. Instead, NetCoMi uses the adjusted Rand index [101]. The adjusted values lie in the range $[-1,1]$, where 1 corresponds to identical clusterings and 0 to the expected value for two random clusterings. Consequently, positive index values imply that two clusterings are more similar and negative values less similar than expected at random. Following [102], NetCoMi uses a permutation procedure to test whether a calculated value is significantly different from zero. However, this test does not signify whether the clusterings are significantly different between the groups. Details about this approach and its implementation in R are given in Supplementary material 3.3.

### Differential association analysis

NetCoMi also allows to test differences between the estimated associations themselves rather than network properties. This analysis is referred to as *differential association analysis*. Table 5 shows the three approaches available in NetCoMi (Supplementary material 4 for details).

**Table 5:** Test procedures for identifying differential associations available in NetCoMi. Fisher’s z-test and the Discordant method are based on correlations that are Fisher-transformed into z-values. These two methods are thus not suitable for the other association measures included in NetCoMi.

**Test method**	**Originally designed for...**	**In NetCoMi used for...**
Fisher’s z-test	Correlation	Correlation
Non-parametric test [105]	“connectivity scores”[105]: $\bullet $ Correlation $\bullet $ Partial correlation $\bullet $ Partial least squares based scores	$\bullet $ Correlation $\bullet $ Proportionality (due to its similarity to correlations) $\bullet $ Conditional dependence / partial correlations
Discordant method [106]	Correlation	Correlation

Fisher’s z-test [103] is a common method for comparing two correlation coefficients, assuming normally distributed *z* values (the transformed estimated correlations) and thus a correct specification of their variance. Novel approaches without normality assumption have been proposed [104], but are restricted to Pearson correlations. Therefore, we implemented a resampling-based procedure [105] as non-parametric alternative, which is applicable to association measures other than correlation. The Discordant method [106] as the third available method is also based on Fisher’s z values, but groups correlations with a similar magnitude and direction based on mixture models.

These approaches enable the construction of a differential network, where only differentially associated taxa are connected. More precisely, two taxa are connected if their association is either significantly different between the two groups (to a user-defined significance level) or identified as being different by the Discordant method.

## Application of NetCoMi on a real data set

We use data from the GABRIEL Advanced Surveys (GABRIELA) [107] to illustrate the application of NetCoMi version 1.0.1 [108]. GABRIELA is a multi-center study, carried out in rural areas of southern Germany, Switzerland, Austria and Poland, which provides new insights into the causes of the protective effect of exposure to farming environments for the development of asthma, hay fever and atopy [107]. The study comprises the collection of biomaterial and environmental samples including mattress dust from children’s rooms and nasal swabs, for which 16S rRNA amplicon sequencing data are available. After some preprocessing steps (Table S4), a total of $p=707$ bacterial genera remain for mattress dust for a subset of $N=1022$ subjects. The nasal data set consists of $p=467$ genera for $N=1033$ subjects.

**Figure 3:**
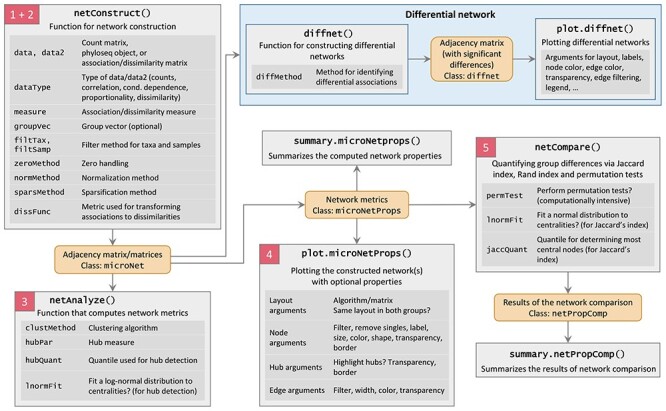
Main NetCoMi functions. For each function, its purpose together with its main arguments is shown. The objects returned from the respective functions are colored in orange. The steps (colored in red) correspond to the steps of the overall workflow shown in Figure 1.

The NetCoMi functions corresponding to the main steps of our proposed workflow (Figure 1) are netConstruct() for network construction (Section 2.3), netAnalyze() for network characterization (Section 2.4), and netCompare() for network comparison (Section 3). An overview of the exported (and thus usable) NetCoMi functions together with their main arguments is given in Figure 3. In the following, we use the SPRING method as a measure of partial correlation for constructing exemplary microbial association networks and Aitchison’s distance as a dissimilarity measure for constructing sample similarity networks. The construction of a differential network is described in Supplementary material 5.5. We also demonstrate the integration of external networks into NetCoMi’s workflow using BAnOCC-derived microbial association networks [109] (Supplementary material 6). In Supplementary material 7, we investigate the variability of microbial networks among different network construction methods. The results reveal strong differences between the networks based on different association measures as well as different normalization methods whereas the zero replacement methods available in NetCoMi lead to quite similar networks, if the remaining arguments are fixed.

### Constructing a single microbial network

In principle, the netConstruct() function allows the specification of any combination of methods for association estimation, zero treatment and normalization. However, since NetCoMi is mainly designed to handle compositional data, a warning is returned if a chosen combination is not compositionally aware.

**Figure 4:**
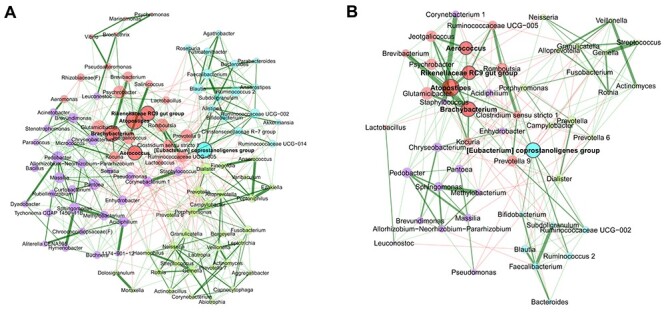
Bacterial associations for the combined data set with samples from Ulm and Munich. The SPRING method [55] is used as association measure. The estimated partial correlations are transformed into dissimilarities via the “signed” distance metric and the corresponding (non-negative) similarities are used as edge weights. Green edges correspond to positive estimated associations and red edges to negative ones. Eigenvector centrality is used for defining hubs (nodes with a centrality value above the empirical 95% quantile) and scaling node sizes. Hubs are highlighted by bold text and borders. Node colors represent clusters, which are determined using greedy modularity optimization. **A:** complete network for the data set with 100 taxa and 1022 samples. Unconnected nodes are removed. **B:** reduced network, where only the 50 nodes with the highest degree are shown. Centrality measures and clusters are adopted from the complete network.

To generate the network shown in Figure 4A, we pass the combined count matrix containing dust samples from Ulm and Munich to netConstruct(). Filter parameters are set in such a way that only the $100$ most frequent taxa are included in the analyses leading to a $1022 \times 100$ read count matrix for our data. Depending on the association measure, a method for zero handling, normalization and sparsification could be chosen (Figure S1 and Table S1). Since these steps are already included in SPRING, they are skipped in our example. If associations have already been estimated in advance, the external association matrix can be passed to netConstruct() instead of a count matrix. In this mode, the user only needs to define a sparsification method and a dissimilarity function.

Besides the available options for transforming the associations into dissimilarities (Supplementary Figure S1), the function also accepts a user-defined dissimilarity function. The choice of dissimilarity function also influences the handling of negative associations. In Figure 4, we use the “signed” distance metric, where strong negative correlations lead to a high dissimilarity (and thus to a low edge weight in the adjacency matrix). Figure S4 shows a network plot where the “unsigned” metric is used.

netConstruct() returns an object of class microNet, which can be directly passed to netAnalyze() to compute network properties (Table S5). Applying the plot function to the output of netAnalyze() leads to a network visualization, as shown in Figure 4A. In this plot, network characteristics are emphasized in different ways: hubs are highlighted, clusters are marked by different node colors, and node sizes are scaled according to eigenvector centrality. Alternatively, node colors and shapes can represent features such as taxonomic rank. Node sizes can be scaled according to other centrality measures or absolute/relative abundances of the corresponding taxa. Node positions are defined using the Fruchterman-Reingold algorithm [110]. This algorithm provides a force-directed layout aimed at high readability of the network by placing pairs of nodes with a high absolute edge weight close together and those with low edge weight further apart.

The plot() method implemented in NetCoMi includes several options for selecting nodes or edges of interest to facilitate the readability of the network plot without influencing the calculated network measures. In Figure 4B, for instance, we display the 50 bacteria with highest degree, facilitating network interpretability.

NetCoMi identified four clusters and the following five hub nodes: *Aerococcus*, *Atopostipes*, *Brachybacterium*, *Rikenellaceae RC9 gut group* and *[Eubacterium] coprostanoligenes group*. The strongest positive association is between *Ezakiella* and *Peptoniphilus* with a partial correlation of 0.55 (in the green cluster in Figure 4A). The strongest negative correlation is -0.09 between *Alistipes* (in the blue cluster) and *Lactobacillus* (in the red cluster).

### Comparing networks between two study centers

**Figure 5:**
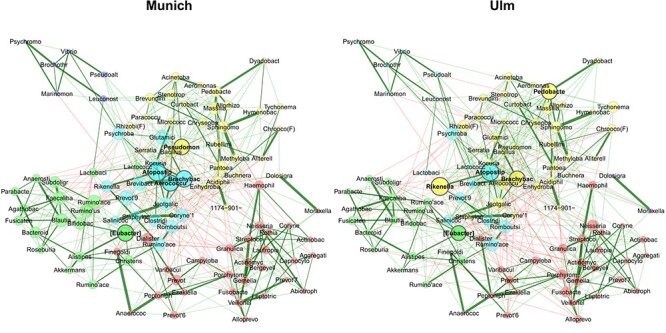
Comparison of bacterial associations in the mattress dust between the study centers Munich and Ulm. The SPRING method [55] is used as association measure. The estimated partial correlations are transformed into dissimilarities via the “signed” distance metric and the corresponding similarities are used as edge weights. Eigenvector centrality is used for defining hubs and scaling node sizes. Node colors represent clusters, which are determined using greedy modularity optimization. Clusters have the same color in both networks if they share at least two taxa. Green edges correspond to positive estimated associations and red edges to negative ones. The layout computed for the Munich network is used in both networks. Nodes that are unconnected in both groups are removed. Taxa names are abbreviated (Table S9 for the original names).

For network comparison, the combined count matrix is again passed to netConstruct(), but this time with an additional binary vector assigning the samples to one of the two centers. This leads to the network plots shown in Figure 5 (Table S7 for the summary of network properties). The layout computed for the Munich network is used for both networks to facilitate the graphical comparison and making differences clearly visible. We observe only slight differences in the estimated associations. Furthermore, both networks show similar clustering and agree on three (out of five) hub nodes: *Atopostipes*, *Brachybacterium* and *[Eubacterium] coprostanoligenes group*.

The quantitative comparison is done by passing the R object returned from netAnalyze() to netCompare(). Comparisons of all global measures included in NetCoMi and the five genera with the highest absolute group difference for degree and eigenvector centrality, respectively, are given in Table 6. Supplementary Table S8 extends the output to the 10 genera with the highest absolute group difference, and also includes betweenness and closeness centrality. For none of the four centrality measures, any significant differences are observed, confirming the descriptive analyses. *Pseudomonas*, *Pedobacter* and *Rikenellaceae RC9 gut group* as hub taxa in only one of the networks show high differences in eigenvector centrality. However, the differences are not deemed significant (Table 6). Global network properties (upper part of Table 6) are also not significantly different for $\alpha = 0.05$.

**Table 6:** Results from testing global network metrics and centrality measures of the networks in Figure 5 for group differences (via permutation tests using 1000 permutations). Shown are respectively the computed measure for Munich and Ulm, the absolute difference, and the P-value for testing the null hypothesis $H_0: |\textrm{diff}| = 0$. For the centrality measures, P-values are adjusted for multiple testing using the adaptive Benjamini-Hochberg method [73], where the proportion of true $H_0$ is determined according to Langaas et al. [74]. For degree and eigenvector centrality, the five genera with the highest absolute group difference are shown. The centralities are normalized to [0,1] as described in Table 4. Highly different eigenvector centralities (even if not significant) describe bacteria with highly different node sizes in the network plots in Figure 5 such as *Pseudomonas*, which is a hub in Munich and much less important in Ulm.

	**Munich**	**Ulm**	**abs. diff.**	***P*-value**
Global network measures:	
Average path length	1.576	1.555	0.022	0.60440
Clustering coefficient	0.279	0.249	0.029	0.11189
Modularity	0.458	0.407	0.051	0.08192.
Vertex connectivity	3	4	1	0.68232
Edge connectivity	3	4	1	0.68132
Density	0.103	0.100	0.003	0.55045
Degree (weighted):	
Corynebacterium 1	0.162	0.071	0.091	0.19905
Rikenellaceae RC9 gut group	0.101	0.192	0.091	0.82936
Lactobacillus	0.121	0.051	0.071	0.82936
Pedobacter	0.101	0.172	0.071	0.82936
Pseudomonas	0.202	0.131	0.071	0.99622
Eigenvector centrality:	
Pseudomonas	0.852	0.417	0.435	0.57458
Rikenellaceae RC9 gut group	0.595	1.000	0.405	0.57458
Ruminococcaceae UCG-002	0.767	0.379	0.389	0.57458
Corynebacterium 1	0.670	0.290	0.379	0.57458
Pedobacter	0.526	0.873	0.346	0.83396

Significance codes: $^{***}$: 0.001, $^{**}$: 0.01, $^{*}$: 0.05, $\boldsymbol{.}$: 0.1

Table 7 summarizes Jaccard indices expressing the similarity of sets of most central nodes and the hub nodes among the two centers. They do not imply any group differences, which would be indicated by a small probability P($J\le j$). Similarly, the adjusted Rand index $(0.752, \text{P-value}=0)$ indicates a high similarity of the two clusterings, which is also highlighted in Figure 5.

**Table 7:** Jaccard index values corresponding to the networks shown in Figure 5. Index values $j$ express the similarity of the sets of most central nodes and also of the sets of hub taxa between the two networks. “Most central” nodes are those with a centrality value above the empirical 75% quantile. Jaccard’s index is 0 if the sets are completely different and 1 for exactly equal sets. $P(J\le j)$ is the probability that Jaccard’s index takes a value less than or equal to the calculated index $j$ for the present total number of taxa in both sets ($P(J\ge j)$ is defined analogously).

	$j$	P($J\le j$)	P($J\ge j$)
Degree	0.323	0.53375	0.61683
Betweenness centr.	0.389	0.81277	0.29332
Closeness centr.	0.471	0.96735	0.06729.
Eigenvec. centr.	0.429	0.91326	0.15482
Hub taxa	0.429	0.82670	0.42936

Significance codes: $^{***}$: 0.001, $^{**}$: 0.01, $^{*}$: 0.05, $\boldsymbol{.}$: 0.1

### Sample similarity networks

**Figure 6:**
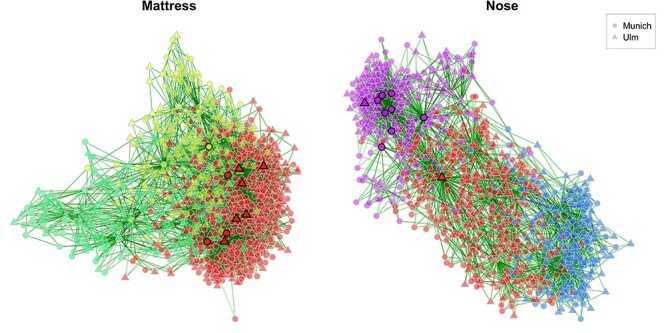
Comparing dissimilarity networks based on Aitchison’s distance [96] (Supplementary Table S3) between mattress dust and nasal swabs for the same set of subjects (nodes). Only samples and taxa with at least 1000 reads, respectively, are included leading to $p_1$=707 genera in the Mattress group, $p_2$=184 genera in the Nose group, and $n$=980 samples in both groups. Counts are normalized to fractions and – since zeros must be replaced for the clr transformation – “multiplicative imputation” ( Table 3 in the main text) is used for zero handling. The dissimilarity matrix is scaled to [0,1] and sparsified using the k-nearest neighbor method ($k$=3 for both networks). Node colors represent clusters, identified using hierarchical clustering with average linkage. A cluster has the same color in both networks if they have at least 100 nodes in common (the minimum cluster size among both groups is 560). Hubs (highlighted by bold borders) are nodes with an eigenvector centrality larger than the 99% quantile of the empirical quantile of eigenvector centralities. Edge thickness corresponds to similarity values (calculated by $1-distance$). Nodes are placed further together, the more similar their bacterial composition is. Whether a sample has been collected in Munich or Ulm is marked by node shapes. Unconnected nodes are removed.

We next consider sample similarity networks from mattress dust and nasal swabs of the same subjects ($N=980$). The process of constructing and comparing sample similarity networks is analogous to the one for association networks. Figure 6 shows the two networks using Aitchison’s distance.

NetCoMi’s quantitative network analysis (Tables S10–S13) reveals strong differences between these networks. The sets of most central nodes are significantly different for all four centrality measures (shown by Jaccard’s index). Hub nodes are also completely different (Jaccard index of zero). We also observe several significantly different global network properties (Table S12). Furthermore, the node’s degree, betweenness and closeness centrality values differ significantly between the groups for several subjects (Table S13), implying that a single subject plays a different role dependent on the investigated microbial habitat.

Clustering analysis broadly identifies three sample groups for mattress dust and nasal swab samples, respectively. In Figure 6, we highlight the partial overlap between the two clustering solutions via color coding. Clusters that have at least 100 nodes in common are plotted by matching colors. This reveals that the red cluster, seen in both networks, shares similar samples across the two habitats.

NetCoMi’s plotting functionality also allows to draw nodes in different shapes, corresponding to additional categorical covariate information about the samples. For instance, in Figure 6, the two node shapes correspond to the two different study centers (Ulm and Munich). This feature could potentially highlights confounding of groups of samples and available covariates. For our example here, however, we observe no noticeable pattern in the clusters with respect to study center.

## Discussion

### Why use NetCoMi?

With NetCoMi we offer an easy-to-use and versatile, integrative R package for the construction, analysis and comparison of microbial networks derived from MGS data. Our package provides a wide variety of compositionally aware association measures, including SparCC [27], proportionality [54], SPIEC-EASI [63] and SPRING [29, 55]. The latter method also enables the analysis of recent quantitative microbiome data sets when both amplicon and quantitative cell count or spike-in control data are available. NetCoMi also incorporates standard association measures, thus widening the scope of the package beyond applications to compositional data, and it connects to the popular WGCNA package [52], enabling principled *soft-thresholding* of correlations and dissimilarity transformations based on topological overlap. The package includes a dedicated list of methods for handling excess zeros in the count matrix and for data normalization in order to account for the special characteristics of the underlying MGS data prior to association estimation.

A unique feature of the NetCoMi framework is its ability to perform differential network and differential association analysis in a statistically principled fashion. Differential network analysis allows not only to uncover the global role of a taxon in the overall network structure but also its changing influence under varying conditions. Differential association analysis [31], on the other hand, can directly assess which associations significantly change across conditions, providing concrete hypotheses for follow-up biological perturbation experiments.

Similar to phyloseq’s [111] plot_net function, NetCoMi also enables network representation and comparison of the MGS data samples themselves, using popular sample dissimilarity or distance measures, such as the Bray-Curtis dissimilarity and the Aitchison distance. Network analysis of the resulting sample-to-sample or subject-to-subject networks can give insights into the heterogeneity of the collected data. For instance, identified *hub subjects* are subjects with “representative” bacterial compositions that may comprise archetypical microbial patterns in the studied population. Sample similarity network analysis thus extends standard sample ordination or cluster analysis, making fine-grain structures of the available microbial sample collection visible.

Whereas methods and tools for the individual analysis steps, such as biological network estimation (e.g. [112, 113] for recent contributions) and (differential) biological network analysis [32–34, 52, 111, 114] are available, NetCoMi offers a unique and modular R software framework that integrates the complete process of estimating, analysing and comparing *microbial* networks. From an end user’s perspective with a specific microbiome dataset and scientific question in mind, NetCoMi will thus facilitate both faster development and reproducibility of the microbial network analysis workflow.

### Which method to choose?

Even though the modular design of NetCoMi allows the user to perform a wide variety of computational workflows, going from the primary data all the way to potentially significant network features, every step of the analysis still warrants careful scientific consideration. For instance, the choice of an association or dissimilarity measure will likely affect all further steps of network analysis and comparison, as we have shown for microbial association networks. However, there is no general consensus in the community about the “right” way to estimate and analyse microbial networks. This is reflected in the heterogeneity of recent simulation studies to assess and compare the performance of compositionally aware as well as traditional association measures. To put some of these studies into context, we give a selection of simulation studies examining the association methods used in NetCoMi in Supplementary Table S17.

Common shortcomings in current simulation studies include the lack of a universal standard (i) to generate realistic synthetic microbial data with a prescribed ground truth, (ii) to perform comparable model selection and (iii) to report generalizable performance metrics. Comparative studies often concentrate on the performance in edge recovery, for instance, via precision-recall curves [28, 55, 115, 116] or distances between true and estimated associations [27, 28, 53, 55]. However, networks derived from penalized marginal correlations (such as SparCC) and partial correlations (such as SPRING) are statistically difficult to compare, thus requiring special modifications which, in turn, limits cross-study comparisons (e.g. Yoon et al. [55] where SparCC correlations are transformed into SparCC partial correlations). Moreover, the sole focus on edge recovery may obfuscate other aspects of correct model recovery, including the shape of degree distributions [28] or detecting hub nodes. For instance, methods with a similar edge recovery precision may greatly vary regarding their ability to determine hub nodes [117]. Thus, if the correct detection of hub nodes is of major interest to the user, present comparative microbial network studies will give little guidance.

Finally, we posit that simulation studies accompanying articles that introduce new methods might also be inherently biased [118, 119]. Neutral comparison studies, i.e. studies that are independent of any new method development [118, 119] are rare in our context or do often not include recently published methods [115]. The main impediment regarding neutral comparison studies is, however, the fact that, to date, no large-scale ground truth network of real biological microbial interactions is available. Such biological gold-standard networks, as available in other contexts (e.g. gene-gene or transcription-factor-gene interactions), would greatly facilitate future comparative studies.

In the absence of a “best method” for microbial network inference and analysis, NetCoMi is intended to give researchers the possibility to apply a consistent and reproducible analysis workflow on their data. Ideally, the selection of the workflow building blocks should be set up once and independent of any hypothesis about the data, thus avoiding the fallacy of starting “fishing” for results that best suit a previously formulated hypothesis. NetCoMi can, however, serve as an ideal tool for principled sensitivity analysis of the inferred results, for instance, by assessing how different normalization and zero handling methods affect the estimated networks, their structural properties and their comparison. Finally, we envision NetCoMi to provide a useful framework for future simulation studies that evaluate and compare the performance of different association measures and network inference tools in a reproducible fashion.

### Current limitations and future developments

The current version of NetCoMi is designed to model networks from a single domain of life, e.g. bacteria, fungi or viruses. However, microbes from different domains of life often share the same habitat and likely influence each other [120]. Joint cross-domain network inference already revealed considerable alterations of the overall network structure and network features, compared to their single-domain counterparts [121]. Extending NetCoMi to cross-domain network analysis is thus an important future development goal. Likewise, environmental factors, such as chemical gradients and temperature, as well as batch effects are known to influence microbial abundances and composition and thus bias network estimation [117, 122, 123]. In the current NetCoMi version, we assume that the user has already corrected the microbiome data for these latent influences. However, several inference methods can directly incorporate known [124, 125] or unknown latent factors [122] into network learning. Including or connecting these approaches with NetCoMi will likely increase the robustness and generalizability of future workflows.

A core feature of NetCoMi is the use of statistical tests at various stages of the computational workflow. For instance, statistical tests can be employed for edge selection in network sparsification. Since statistical power depends on sample size, the sparsified structure of a network will likely depend on the number of available samples. A comprehensive understanding between number of samples, sparsification and network structure is currently elusive. NetCoMi relies on permutation tests for several statistical tests. The lower limit of *P*-values arising from permutation tests is directly related to the number of available permutations (Section 4). This already required proper adjustment of the calculation for small numbers [126, 127]. For extended simulation studies, NetCoMi’s dependence on permutation tests may prove to be computer intensive. Thus, integration of less demanding alternatives to permutation tests would represent a welcome feature in NetCoMi.

Finally, despite incorporating a comprehensive list of methods in our R package, we do not claim completeness. This can hardly be achieved in the vibrant field of microbiome research where new methods are constantly developed. We alleviated this shortcoming via NetCoMi’s modular structure which allows certain parts of our workflow to be combined with external methods. For instance, users can input a user-defined association or dissimilarity matrix rather than a data matrix, and then proceed with NetCoMi’s standardized network analysis modules.

In summary, we believe that NetCoMi is a useful addition to the modern microbiome data analysis toolbox, enabling rapid and reproducible microbial network estimation and comparison and ideally leading to robust hypotheses about the role of microbes in health and disease [19].

**Key Points**

- Current high-throughput sequencing count data carry only relative or compositional information, thus requiring dedicated statistical analysis methods.
- NetCoMi is a comprehensive R package that implements the complete workflow of constructing, analysing, and comparing microbial association networks.
- NetCoMi integrates an extensive list of methods that take into account the special characteristics of marker-gene and metagenomic sequencing data, including methods for zero count handling, normalization and association estimation.
- The package also offers functionality for constructing sample similarity networks as well as differential networks including appropriate methods for identifying differentially associated taxa.

## Data availability

The data underlying this article will be shared on reasonable request to the corresponding author upon approval by the involved institutional review boards.

## Supplementary Material

NetCoMi_supplement_bbaa290

Rscript_manuscript_bbaa290

Rscript_supplement6_bbaa290

Rscript_supplement7_bbaa290

## Notes

**Stefanie Peschel** is a PhD student at the Institute of Asthma and Allergy Prevention, Helmholtz Zentrum München (Neuherberg, Germany) with background in statistics. Her current research focuses on understanding the role of the human microbiome for the development of asthma and allergies using network analysis methods.

**Christian L. Müller** is group leader at the Institute of Computational Biology, Helmholtz Zentrum München (Munich, Germany), professor at the Department of Statistics, LMU Munich (Germany), and researcher at the Center for Computational Mathematics, Flatiron Institute, New York. His research interests include computational statistics, optimization, and microbiome data analysis.

**Erika von Mutius**, MD MSc, Professor of Pediatric Allergology, is head of the Asthma and Allergy Department at the Dr von Hauner Children’s Hospital in Munich and leading the Institute of Asthma and Allergy Prevention at Helmholtz Zentrum München (Neuherberg, Germany). Her research group is focusing on the determinants of childhood asthma and allergies.

**Anne-Laure Boulesteix** is an associate professor at the Institute for Medical Information Processing, Biometry and Epidemiology, LMU Munich (Germany). Her activities mainly focus on computational statistics, biostatistics and research on research methodology.

**Martin Depner** is a postdoctoral research fellow at the Institute of Asthma and Allergy Prevention, Helmholtz Zentrum München (Neuherberg, Germany) with background in medical biometry. His current research focuses on studies of the microbiome (16S rRNA) and the protective effects of growing up on a farm on asthma and allergies.
