# Supplementary material for: NetCoMi: network construction and comparison for microbiome data in R
Source: Brief Bioinform. 2020 Dec 3;22(4):bbaa290. doi: 10.1093/bib/bbaa290 (PMC8293835; doi:10.1093/bib/bbaa290)
Supplement: NetCoMi_supplement_bbaa290 [file netcomi_supplement_bbaa290.pdf]

NetCoMi: Network Construction and Comparison for Microbiome Data in R

Supplementary Material

Contents

1 Further details on association measures **2**

1.1 Correlation measures . . . 2

1.1.1 SparCC . . . 2

1.1.2 CCLasso . . . 2

1.1.3 CCREPE . . . 2

1.2 Measures of conditional independence . . . 3

1.2.1 SPIEC-EASI . . . 3

1.2.2 SPRING . . . 3

1.2.3 gCoda . . . 3

2 Further details on network construction **4**

2.1 Detailed workflow for determining the adjacency matrix from associations . . . 4

2.2 The WGCNA approach . . . 4

2.2.1 Soft-thresholding . . . 4

2.2.2 Main steps of the WGCNA approach . . . 4

2.3 Notes on normalization and zero handling . . . 6

2.4 Methods for sample and taxa filtering implemented in NetCoMi . . . 6

2.5 Sample similarity networks . . . 7

3 Group comparison of network properties **9**

3.1 Permutation tests - Testing centrality measures and global properties for group differences . . . 9

3.2 Jaccard's index . . . 9

3.3 The adjusted Rand index . . . 9

4 Differential association analysis **10**

4.1 Fisher's z-test . . . 10

4.2 Non-parametric tests . . . 10

4.3 Discordant method . . . 11

4.3.1 Theoretical Background . . . 11

4.3.2 Comparison to other methods . . . 13

5 Case study: Applying NetCoMi to real data from GABRIELA study **14**

5.1 Data preprocessing . . . 14

5.2 Network properties of the single microbial association network . . . 15

5.3 Microbial association network with "unsigned" distance . . . 16

5.4 Additional results for network comparison using SPRING as association measure . . . 18

5.5 Differential network . . . 21

5.6 Comparison of sample similarity networks . . . 22

6 Integrating external networks into NetCoMi **25**

7 Comparison of network construction methods using GABRIELA data **27**

8 Existing simulation studies comparing association and dissimilarity measures **35**

1 Further details on association measures

1.1 Correlation measures

1.1.1 SparCC

SparCC (Sparse Correlations for Compositional data) (Friedman and Alm, 2012) estimates Pearson correlations of the true unobserved abundances via an approximative approach based on the variance of log-ratios $t_{ij} = \text{Var}\left(\log \frac{x_i}{x_j}\right)$. The approximation is valid under the assumptions that the number of taxa is large and that they are sparsely correlated.

Since log-ratios cannot be calculated if the count matrix contains any zeros, they need to be handled suitably. Friedman and Alm (2012) propose a Bayesian approach, where the true (unobserved) fractions, given the observed counts, are assumed to be Dirichlet distributed. All observations are replaced by random samples from that posteriori. The sparsity assumption is reinforced by iterating the process of correlation estimation, where the taxa pair with the highest estimated correlation is excluded in each step as long as their correlations exceed a predefined threshold. Then, the process of estimating fractions and computing correlations is repeated. To account for variation in the sampling process, the whole approach is repeated several times. The authors of SparCC suggest at least 100 repetitions, which is used as default value in `NetCoMi`.

We adopted some code from the `r-sparcc` package (Filosi, 2017) to implement a parallelized version of the SparCC algorithm so that the repetitions of the basic procedure are executed parallel using multiple CPU cores. In `NetCoMi`, the basic algorithm is implemented in C++ (using the R package `Rcpp`) to further reduce run time.

1.1.2 CCLasso

CCLasso (Correlation inference for Compositional data through Lasso) (Fang et al., 2015) is another approach for inferring the latent correlations between microorganisms. In CCLasso, log-ratios are used to overcome compositional effects as well. Furthermore, a sparsity assumption is necessary to infer the covariance matrix, and thus the correlation matrix, from the observed compositional data. The basis of CCLasso is an algorithm for finding a positive definite correlation matrix with elements in [-1,1] by minimizing a loss function plus an l_1-penalty. The loss function considers errors resulting from estimating the covariance matrix from the observed compositional data instead of the true abundances and the penalty incorporates the sparsity assumption. The R code for CCLasso published on GitHub (Fang, 2016) has been adopted to `NetCoMi`.

1.1.3 CCREPE

CCREPE (Compositionality Corrected by REnormalization and PErmutation), originally called “ReBoot method”, has been proposed by Faust et al. (2012). CCREPE operates on the observed relative abundances. CCREPE uses a procedure to test for statistical significance of the estimated correlations that takes into account spurious correlations caused by compositional effects.

The idea is that the null distribution constructed via permutation should incorporate that a part of the observed correlation is caused by compositionality alone. To construct an appropriate permutation distribution, the relative abundances are permuted and re-normalized so that they sum up to one within a sample again. The correlations between organisms are calculated for the permuted data, and this process is repeated to obtain a null distribution which should represent correlations caused by compositionality alone. Then, a bootstrap confidence interval is constructed which is located around the ”observed“ correlation. Finally, a z-test is used to test for differences in the mean of both distributions. A significant result implies that the estimated correlation for the observed data is significantly different from the correlation induced by compositionality. For the CCREPE approach, a zero treatment is possible but not necessarily needed.

As stated in Faust et al. (2012), relative data do not contain all information about the true absolute abundances, which is why it is generally not possible to fully distinguish between spurious correlations due to compositionality from original correlation Faust et al. (2012).

1.2 Measures of conditional independence

1.2.1 SPIEC-EASI

A basic assumption of SPIEC-EASI (Sparse Inverse Covariance Estimation for Ecological Association Inference) (Kurtz et al., 2015) is that the covariance matrix for *clr*-transformed data is approximately equal to the ”true“ basis covariance matrix if the number of taxa p is large. After *clr*-transformation, an undirected weighted graph is estimated using one of two provided methods: neighborhood selection (MB) or sparse inverse covariance selection (GL). In the MB approach, for every single node, a penalized regression model is solved. The GL method, in contrast, seeks to reconstruct the whole graph by solving a global convex optimization problem. In the $p > n$ setting, both methods assume the inverse covariance to be sparse.

The neighborhood selection approach leads to node-wise partial correlations for each node that after symmetrization, can serve as weighted adjacency matrix. The GL approach results in a positive definite inverse covariance matrix (precision matrix) Σ^{-1}, whose negative entries are proportional to partial correlations. The non-zero entries of the matrix form the adjacency matrix. Both methods include a tuning parameter λ controlling the sparsity of the estimates. Model selection via the “Stability Approach to Regularization Selection (StARS)”. Here, random subsamples of the data are taken repeatedly, from which graphs are estimated across the entire regularization path. From the resulting collection of graphs, an overall edge variability statistic is computed across the λ-path. The StaRS-selected λ is the smallest λ at which a user-defined stability threshold is reached.

1.2.2 SPRING

The SPRING (Semi-Parametric Rank-based approach for INference in Graphical model) approach (Yoon et al., 2019) follows a similar workflow as SPIEC-EASI but is applicable both to quantitative and relative abundance data. In SPRING, the correlation matrix is estimated via a semi-parametric rank-based correlation estimator. The data are assumed to follow a truncated Gaussian copula model (Yoon et al., 2018), which extends the original Gaussian copula model (Liu et al., 2012) in order to handle data with excess zeros. The latent correlation matrix is estimated using Kendall’s τ and element-wise univariate optimization of an inverse copula function.

Sparse conditional dependencies are then inferred using the neighborhood selection approach using the latent correlation estimate. Analogous to SPIEC-EASI, model selection is performed via StARS. When applied to compositional data, SPRING introduces a modified clr transformation ($mclr$) that does not require pseudocounts. The $mclr$ transform calculates the geometric mean from positive proportions only, computes the clr for all positive parts with respect to this mean, and applies a positive shift to all non-zero components to ensure strict positivity. Zero proportions remain zero in the $mclr$-transformed vector.

1.2.3 gCoda

The basic assumption of gCoda (Fang et al., 2017) is that the unobserved absolute read counts y follow a multivariate normal distribution with mean μ and covariance matrix Σ, which describes the dependency structures between variables and thus is the term of interest. From $\log(y) \sim N_p(\mu, \Sigma)$ follows that the observed relative counts are logistic normal distributed. In gCoda, also a sparsity assumption is needed to determine the covariance matrix. To satisfy this assumption –analogous to CCLasso– a l_1 penalty is used, which is added to the negative log-Likelihood. The resulting optimization problem is solved via a Majorization-Minimization algorithm to estimate the inverse covariance matrix.

2 Further details on network construction

2.1 Detailed workflow for determining the adjacency matrix from associations

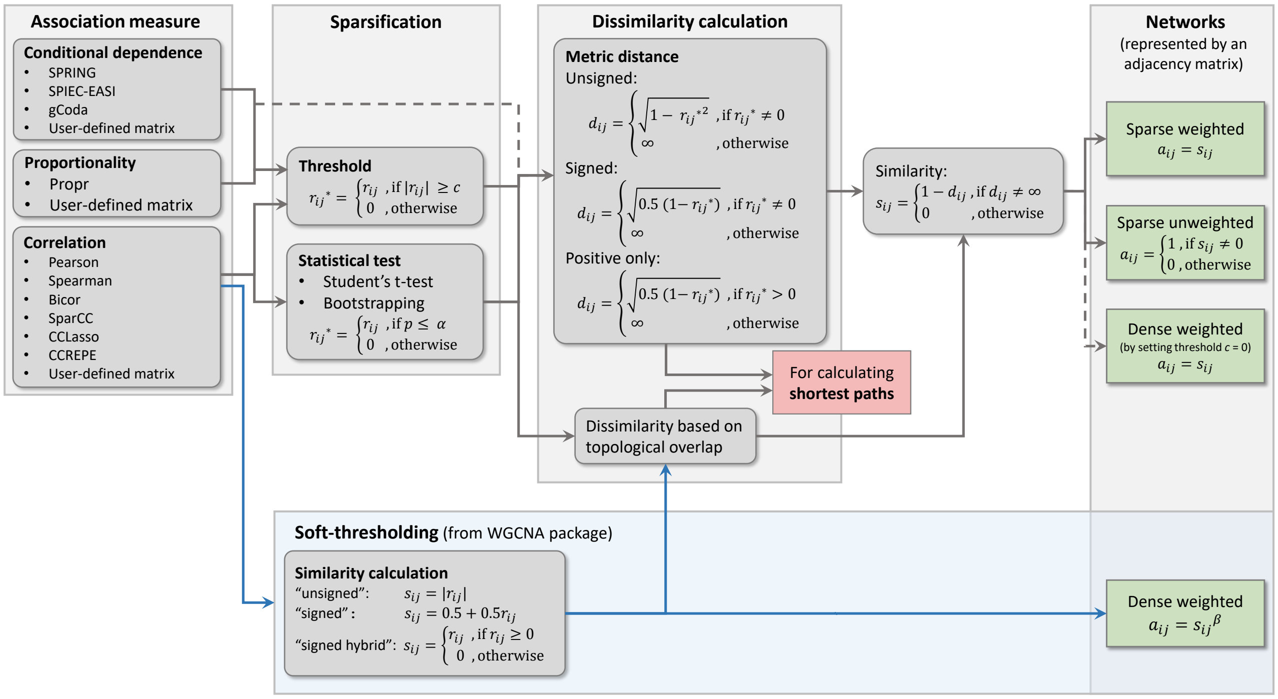

Figure S1: Approaches for constructing the adjacency matrix with entries a_{ij} from estimated associations r_{ij} that are available in NetCoMi. Depending on the association measure, the user can choose between different sparsification methods. The associations are then transformed into dissimilarities d_{ij}, which are used for network properties based on shortest paths. The dissimilarity between nodes with $r^*_{ij} = 0$ is set to infinity so that a direct path exists only between nodes with an association different from zero. The dissimilarities are, in turn, transformed into similarities via $s_{ij} = 1 - d_{ij}$. Similarities are used for similarity-based network properties as well as edge weights in the network plot. For correlations, in addition to a threshold and statistical testing, the soft-thresholding approach from the WGCNA package (Langfelder and Horvath, 2008) is available (blue path). The WGCNA package offers three possibilities for transforming correlation into similarity: "unsigned", "signed", and "signed hybrid", which are also available in NetCoMi. Following Langfelder and Horvath (2008), these similarities are used as edge weights and for similarity-based network measures, whereas for shortest paths a dissimilarity measure based on the topological overlap matrix (TOM) is used. The TOM-based dissimilarity has generally been made available as dissimilarity transformation in NetCoMi.

2.2 The WGCNA approach

2.2.1 Soft-thresholding

WGCNA has originally been proposed by Zhang and Horvath (2005) as "Weighted Gene Co-expression Network Analysis", which later led to the R package WGCNA ("weighted correlation network analysis") (Langfelder and Horvath, 2008). In this approach, a "soft thresholding" method is used for network construction as an alternative to common edge selection methods. The Zhang and Horvath (2005) argue that "hard" thresholding, such as hypothesis testing or a cutoff value, goes along with a loss of information. This is plausible because only selected edges are part of the network, and thus, are included in further analyses such as cluster building or calculating centrality measures. The authors propose to raise the estimated correlation to the power of a predefined value β to determine the adjacencies. For $\beta > 1$, the adjacency increases not linearly anymore. Instead, small correlation values are pushed towards zero so that they become less important in the network. An overview, how connection strength changes for different powers β is given in Mason et al. (2009).

2.2.2 Main steps of the WGCNA approach

The idea of the WGCNA approach (Zhang and Horvath, 2005) is as follows: The estimated correlations between every two variables i and j are transformed into similarities s_{ij}. The entries of the adjacency matrix are then defined as $a_{ij} = s^{\beta}_{ij}$. As the next step, dissimilarity values based on the topological overlap measure (TOM) are determined from the estimated similarities. These dissimilarities are the basis for determining clusters or modules via a hierarchical clustering algorithm.

Thus, in contrast to our proposed workflow (where the correlations are transformed to distances first, which are then, in turn, transformed to similarities), in WGCNA the similarity values are directly calculated from the correlations.

The `WGCNA` package (Langfelder and Horvath, 2008) offers different options for transforming correlations to similarities leading to three network types, which handle negative correlations in different ways. The proposed network types are: "unsigned","signed", and "signed hybrid", with the following corresponding transformations:

$$s_{ij}^{\text{unsigned}} = |r_{ij}|,$$

$$s_{ij}^{\text{signed}} = 0.5 + 0.5\, r_{ij},$$

$$s_{ij}^{\text{signed hybrid}} = \begin{cases} r_{ij}, & \text{for } r_{ij} > 0 \\ 0, & \text{otherwise.} \end{cases}$$

Thus, the estimated correlations, which are located in [-1,1] are transformed into similarities ranging from 0 to 1.

The power β is determined based on a scale-free criterion. The idea is that the degree distribution $P(k)$, which is defined as the fraction of vertices in a network with k adjacent nodes, follows a power-law distribution: $P(k) \sim k^{-\gamma}$ for most biological networks. A linear regression model is used to assess how well $P(k)$ fits a power-law distribution. Networks leading to an $R^2 > 0.8$ are assumed to be approximately scale-free (Zhang and Horvath, 2005). To choose an appropriate power β, the authors suggest generating the adjacency matrix for different β values and choose the smallest power for which the scale-free topology is fulfilled. This can be done in R via the `pickSoftThreshold` function (Langfelder and Horvath, 2008). Alternatively, a user-defined power parameter is accepted as input.

We adopted the aforementioned approaches of the soft-thresholding approach from the `WGCNA` package to `NetCoMi` via own `R` code. Thus, the user of our package can choose between one of the options "signed", "unsigned" and "signed hybrid" to generate similarities, which are transformed to adjacencies via s_{ij}^{β}. The power parameter can either be defined in advance or computed automatically. In the latter case, the `pickSoftThreshold()` function is used. Following the WGCNA approach Zhang and Horvath (2005), TOM-based dissimilarities are used as the basis for clustering, but also for calculating shortest paths.

2.3 Notes on normalization and zero handling

Table S1: Overview of association measures implemented in `NetCoMi`. For each measure is shown, whether it is suitable for read count data without transforming the data in advance, whether a zero treatment is necessary and which normalization is accepted in `NetCoMi`. The function for network construction returns a warning when the chosen combination of association measure and normalization method may lead to compositional effects.

Measure		Compositionally aware?	Zero treatment	Normalization
Pearson coef.	Correlation	no	not needed	any
Spearman coef.		no	not needed	any
Biweight midcorr.		no	not needed	any
SparCC		yes	included	none
CCLasso		yes	needed	fractions
CCREPE		yes	not needed	fractions
ρ	Proportionality	yes	needed	none
SPRING	Conditional dependence	yes	included	none
SPIEC-EASI		yes	included	none
gCoda		yes	needed	fractions
Euclidean distance	Dissimilarity / distance	no	not needed	any
Bray-Curtis dissim.		no	not needed	any
Kullback-Leibler Divergence (KLD)		no	needed	any
Jeffrey divergence		no	needed	any
Jensen-Shannon diverg.		no	needed	any
Compositional KLD		yes	needed	fractions
Aitchison distance		yes	needed	fractions

2.4 Methods for sample and taxa filtering implemented in NetCoMi

Table S2: Methods for preprocessing the count matrix by filtering samples and taxa implemented in `NetCoMi`. Except for `highestFreq` and `highestVar`, several methods can be combined. The parameter x is user-defined.

**Argument name**	**Description**
Sample filtering:	
`totalReads`	Keep samples with a total number of reads of at least x.
`numbTaxa`	Keep samples for which at least x taxa are observed.
`highestFreq`	Keep the x samples with highest frequency.
Taxa filtering:	
`totalReads`	Keep taxa with a total number of reads of at least x.
`relFreq`	Keep taxa whose number of reads is at least x% of the total number of reads.
`numbSamp`	Keep taxa observed in at least x samples.
`highestVar`	Keep the x taxa with highest variance.
`highestFreq`	Keep the x taxa with highest frequency.

2.5 Sample similarity networks

`NetCoMi` provides the possibility to use a dissimilarity measure for network construction instead of association measures. The available measures are shown in Table S3. Using one of these measures leads to a network where nodes are subjects/samples instead of taxa. Aitchison's distance, the compositional Kullback-Leibler divergence (KLD) as well as the Bray-Curtis index are developed for quantifying differences in biological communities. In particular, the Aitchison distance and compositional KLD are designed for compositional data, but also the Bray-Curtis dissimilarity is appropriate for the application on compositional data (Weiss et al., 2016).

The complete workflow for constructing a dissimilarity-based network is shown in Figure S2. Data preparation including filtering, zero handling, and normalization is on the whole similar to that of association networks. The estimated dissimilarity matrix with entries d_{kl} expressing the dissimilarity between two samples k and l is a $n \times n$ matrix with entries in [0,1] or $[0, \infty)$, depending on the dissimilarity measure. In the latter case, the dissimilarities are scaled to [0,1] by

$$d_{kl} = \frac{d_{kl} - min(d)}{max(d) - min(d)},$$

where d is the vector with dissimilarities of all pairs of subjects (Han et al., 2011).

Two sparsification methods are available in `NetCoMi`: (i) specifying a threshold so that subjects with a dissimilarity below this value are connected, and (ii) The k-nearest neighbors (KNN) algorithm, where a vertex v_i is connected to the k vertices with minimum dissimilarity to v_i Marchette (2005).

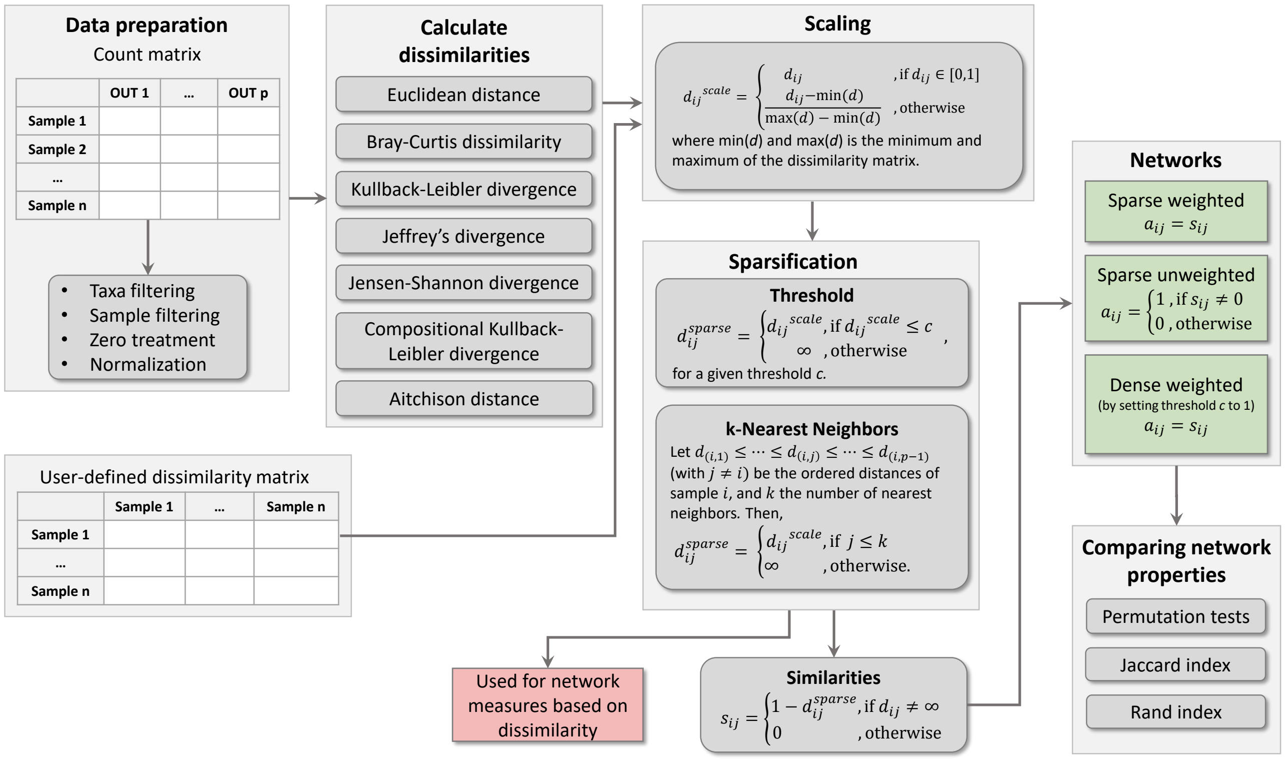

Figure S2: Our proposed workflow for constructing, analyzing, and comparing networks based on dissimilarity between samples. If none of the provided dissimilarity measures should be used, a dissimilarity matrix is accepted as input. If the dissimilarity matrix contains values larger than 1, the values are scaled to [0,1] to facilitate the definition of the threshold (if "threshold" is used as a sparsification method) and to improve the graphical output. The `nng()` function from the `cccd` package (Marchette, 2015) is used for constructing a network with k-Nearest Neighbors as a sparsification method. The function implements the approach proposed by Marchette (2005) with an option for constructing a mutual KNN network, where the nodes must be neighbors of each other. The scaled dissimilarities $D(x_k, x_l)^{scale}$ between subjects k and l are used for network measures based on shortest paths. The corresponding similarities (calculated by $1 - D(x_k, x_l)^{scale}$) are used for measures based on connection strength as well as edge weights in the network plot. The network properties of the two networks can finally be compared using the same methods as provided for association networks.

Table S3: Measures of dissimilarity between two compositions x_k and x_l that are implemented in the `NetCoMi` package. These measures are designed for measuring dissimilarities between samples. Thus, in a network based on one of these measures nodes represent subjects or samples instead of taxa.

**Dissimilarity measures**	**Characteristics**
**Euclidean distance**<br>$D_{\text{Eucl}}(x_k, x_l) = \left[\sum_{i=1}^{p}(x_{ki} - x_{li})^2\right]^{\frac{1}{2}}$	• symmetric with values in [0, ∞)<br>• R: `dist()` (`stats` package)
**Aitchison distance** (Aitchison, 1992)<br>$D_{\text{Ait}}(x_k, x_l) = \left[\sum_{i=1}^{p}(\log(\frac{x_{ki}}{g(x_k)}) - \log(\frac{x_{li}}{g(x_l)}))^2\right]^{\frac{1}{2}}$<br>($g(x) = (\prod_{i=1}^{p} x_i)^{\frac{1}{p}}$ is the geometric mean of composition x)	• Euclidean distance between clr-transformed compositions<br>• symmetric with values in [0, ∞)<br>• suitable for application on compositional data
**Kullback-Leibler divergence** (KLD)<br>(Kullback and Leibler, 1951)<br>$D_{\text{KLD}}(P \Vert Q) = \sum_{i=1}^{p} P(x_i) \log\left(\frac{Q(x_i)}{P(x_i)}\right)$<br>($P$ and Q are the probability distribution functions of x_k and x_l, respectively)<br>$\Downarrow$<br>symmetric version (Johnson and Sinanovic, 2001):<br>$D_{\text{symKLD}}(P, Q) = \frac{1}{2}(D_{\text{KLD}}(P \Vert Q) + D_{\text{KLD}}(Q \Vert P))$	• divergence between two probability distribution functions<br>• asymmetric with values in [0, ∞) ($D_{\text{KLD}}(P \Vert Q) \neq D_{\text{KLD}}(Q \Vert P)$)<br>• since an asymmetric measure leads to a directed network, only the symmetric version is used in `NetCoMi`<br>• R: `KLD()` (`LaplacesDemon` package)
**Jeffrey's divergence** (Jeffreys, 1948)<br>$D_{\text{Jeff}}(P, Q) = D_{\text{KLD}}(P \Vert Q) + D_{\text{KLD}}(Q \Vert P)$	• based on KLD<br>• symmetric with values in [0, ∞)<br>• R: own implementation based on `KLD()`
**Jensen-Shannon divergence** (JSD) (Endres and Schindelin, 2003)<br>$D_{\text{JSD}}(P, Q) = \frac{1}{2}D_{\text{KLD}}(P \Vert M) + \frac{1}{2}D_{\text{KLD}}(Q \Vert M)$<br>with $M = \frac{1}{2}(P + Q)$	• based on KLD<br>• symmetric with values in [0, ∞)<br>• R: own implementation based on `KLD()`
**Compositional Kullback-Leibler divergence (cKLD)**<br>(Martín-Fernández et al., 1999)<br>$D_{\text{cKLD}}(x_k, x_l) = \frac{p}{2}\log(A(x_k/x_l) \cdot A(x_l/x_k)),$<br>where $A(x_k/x_l)$ denotes the arithmetic mean of the vector of ratios $x_k/x_l = \left(\frac{x_{k_1}}{x_{l_1}}, ..., \frac{x_{k_p}}{x_{l_p}}\right)$	• symmetric with values in [0, ∞)<br>• R: own implementation<br>• suitable for application on compositional data
**Bray-Curtis dissimilarity** (Bray and Curtis, 1957)<br>$D_{\text{Bray}}(x_k, x_l) = \sum_{i=1}^{p} \frac{\lvert x_{ki} - x_{li}\rvert}{x_{ki} + x_{li}}$	• symmetric with values in [0,1]<br>• R: `vegdist()` (`vegan` package)

3 Group comparison of network properties

3.1 Permutation tests - Testing centrality measures and global properties for group differences

Centrality measures considered in `NetCoMi` are degree, betweenness, closeness, and eigenvector centrality. To test for each taxon whether these centrality values are significantly different between the two groups, a non-parametric permutation test is performed, which is a common procedure to test graph measures for group differences (Uehara et al., 2014; Hosseini et al., 2012). Own `R` code has been written, which implements the following test procedure.

First, the measures are calculated for the two original adjacency matrices to receive a centrality value for each taxon in both groups. In each repetition, the observed read counts of each subject are then randomly reassigned without replacement to one of the two groups so that the number of subjects within each group equals that of the original data set. For these permuted count data, the association matrices are estimated using the same settings for selecting edges as for the original data. Centrality measures are calculated for the resulting adjacency matrices and finally, the absolute difference between the groups is taken for each measure.

This procedure is repeated many times (1000 by default as suggested by Marozzi (2004)) resulting in a distribution of absolute differences under the null hypothesis that the centrality value is equal in both groups. For each measure, the p-value is calculated as the proportion of permutations in which the absolute difference is greater than or equal to that belonging to the original grouping. A p-value below the desired significance level α means that the observed group difference of the property is significantly higher than expected by chance.

Since n hypothesis tests are conducted simultaneously (where n is the number of nodes), adjusting the permutation p-values for multiple testing is required. As mentioned in Section 2.2 in the main text, two methods for multiple testing adjustment in the case of arbitrary dependencies between the test statistics (which is assumed in our case) are: (1) controlling the local false discovery rate (FDR) as proposed by Efron (2005), and (2) the adaptive Benjamini-Hochberg method (Benjamini and Hochberg, 2000), where the proportion of true null hypotheses is estimated using convex decreasing density estimation according to (Langaas et al., 2005). Both approaches have an increased power compared to the common Benjamini-Yekutieli (Benjamini and Yekutieli, 2001) method, which controls the FDR under dependence as well. Since the number of tests has to be large (several hundred) for method (1), we suggest using method (2) for the permutation tests described in this section.

We implemented the same procedure to test whether the global network properties (average path length, clustering coefficient, modularity, edge and vertex connectivity, and density) are significantly different between the two groups of interest.

3.2 Jaccard's index

In `NetCoMi`, the Jaccard index (Jaccard, 1908) is used for expressing how different the sets of most central nodes are between the two groups. It can be defined via

$$J = \frac{|C_1 \cap C_2|}{|C_1 \cup C_2|} = \frac{C_{12}}{C_1 + C_2 - C_{12}} := \frac{C_{12}}{M},$$

where C_1 is the number of species being most central in group 1, C_2 the number of nodes that are most central in group 2, and C_{12} the number of species that are most central in both groups (Real and Vargas, 1996).

Following Real and Vargas (1996), we implemented an approach to test whether the observed value for Jaccard's index is significantly different from that expected at random. For this purpose, a distribution of all possible Jaccard values for a specific M can be gained by determining all possible distributions of the M species among the sets C_1, C_2 or C_{12}, and calculating the corresponding Jaccard values (Real, 1999). A table with critical values of the Jaccard index has been published (Real, 1999), which could be used for interpreting the output of `NetCoMi`. However, to facilitate the application of `NetCoMi`, the function for network comparison returns p-values, which are calculated according to the explanations in Real and Vargas (1996). For each Jaccard value J, two p-values are returned, which correspond to the hypotheses that J is greater than and smaller than expected at random.

3.3 The adjusted Rand index

The Rand index (Rand, 1971) is calculated as

$$R = \frac{a + b}{\binom{n}{2}},$$

where n is the number of taxa (which is equal in both networks), a is the number of pairs of taxa that are assigned to the same module in both clusterings, and b denotes the number of pairs belonging to different modules in both clusterings.

Qannari et al. (2014) proposed an approach to test whether the adjusted Rand index (ARI) is significantly larger than zero, which is the expected value for two random clusterings. They introduce a permutation procedure to generate an empirical distribution of the adjusted Rand index under the null hypothesis that there is no association between the two

partitions. For this purpose, a large amount of partition pairs has to be produced with the same structure as the original clustering. More precisely, for each partition the number of groups – and within these groups, the number of elements – should be equal to those of the corresponding original partition. This can be achieved by randomly shuffle the module labels for each partition (Qannari et al., 2014).

Finally, for each generated pair of partitions, the adjusted Rand index is calculated and their arithmetic mean $\overline{ARI}$ and Variance $Var(ARI)$ are calculated. These are used to transform the ARI values into a normalized version:

$$NARI = \frac{ARI - \overline{ARI}}{\sqrt{Var(ARI)}},$$

which is determined for each permutation. Also, the index value for the original partition pair (denoted by ARI_0) is transformed into $NARI_0 = \frac{ARI_0 - \overline{ARI}}{\sqrt{Var(ARI)}}$. Based on these results, the p-values are calculated as the proportion of $NARI$ values being larger than the observed value $NARI_0$ (Qannari et al., 2014).

We implemented the procedure in `R` as follows. Clusters are generated via `cuttreeDynamic()` (`dynamicTreeCut` package (Langfelder et al., 2016)). The function returns for each clustering a vector of numerical labels, which assigns each taxon to a cluster. Using `permute()` from the `gtools` package (Warnes et al., 2018) the labels of the two partitions are randomly reassigned and the adjusted Rand index is calculated for the generated clusterings, respectively. Eventually, the normalized index $NARI$ is calculated for all permutations and the original clusterings, and the p-value is calculated as described above. If the determined p-value lies below the desired significance level, the adjusted Rand index for these two clusterings is greater than zero, that is, significantly larger than for two randomly picked partitions.

4 Differential association analysis

From the association measures considered in our work, correlation is the only one for which we have found parametric approaches to test for group differences. Thus, we concentrate on correlations for the moment.

A naive approach to the identify so-called "differential correlations" could be testing if the difference of two estimated correlations is significantly different from zero. This is problematic because the correlation values are only an estimation of the true, unknown correlation coefficient (Fisher, 1915, 1921). Fisher (1915) has shown that shape and variance of their distribution depend on the true correlation value. More precisely, the distribution of the sample correlations r is more skew, the more the true correlation coefficient ρ differs from zero (Fisher, 1921). Thus, the correlations of two populations, which are the two groups of interest in our case, are not directly comparable. To solve this issue, Fisher (1921) suggested transforming the observed values r via $z = \text{arctanh}(r) = \frac{1}{2}\log\frac{1+r}{1-r}$ into Fisher's z-values. These are approximately normally distributed, with standard deviation $\sigma = \frac{1}{\sqrt{n-3}}$ (n denotes the sample size) and thus are suitable for hypothesis testing.

4.1 Fisher's z-test

Fisher's z-test (Fisher, 1992) is a common parametric approach to test the hypothesis $H_0 : \rho_A = \rho_B$ vs. $H_1 : \rho_A \neq \rho_B$ for independent groups A and B. First, the sample correlations r_A and r_B are transformed into Fisher's z-values z_A and z_B. The aforementioned null hypothesis can be rewritten to $H_0 : \rho_A - \rho_B = 0$, which can be tested using the test statistic

$$Z = \frac{(z_a - z_B)}{\sqrt{\frac{1}{n_A-3} + \frac{1}{n_B-3}}},$$

where n_A and n_B denote the sample size of the two groups. Under the null hypothesis, the Z statistic is standard normal distributed so that standard p-values can be calculated via $p = 2(1 - \mathbb{P}(Z \leq |z|))$.

We use our own `R` implementation for testing whether the correlation coefficients are significantly different between the groups. For multiple testing adjustment, all methods provided by `p.adjust()` (`stats` package) as well as a method for controlling the local false discovery rate provided by the `fdrtool` package (Klaus and Strimmer., 2015) are available in `NetCoMi`.

4.2 Non-parametric tests

As an alternative to parametric tests, a nonparametric resampling-based procedure has been added to `NetCoMi`. The idea for this approach is adapted from Gill et al. (2010, 2014), where taxa in biological networks are tested for differential association between two experimental conditions. Their approaches enable testing whether a single gene is differentially associated with all other genes or whether a set of genes is differentially associated in the two networks. We adopted the procedures to implement a non-parametric approach to test if a pair of taxa is differentially associated, which is a special case of their methods. Own `R` code has been written to make the nonparametric test procedure available in `NetCoMi`.

The authors introduce so-called "connectivity scores" (denoted by s in the following), which represent different types of association (e.g. correlation) between two variables i and j. To test whether these variables are differentially associated between networks A and B, the distance

$$d(s_{ij}^A, s_{ij}^B) = |s_{ij}^A - s_{ij}^B|$$

is calculated for the observed data first. Then, the two count matrices are combined to a $(m_A + m_B) \times n$ matrix X containing the read counts for both groups. The row numbers are then randomly shuffled to create a permuted matrix X^*, which is split again into two groups so that the first m_A rows are assigned to the first group and the last m_B rows to the second. Associations are then determined for the permuted groups and the distances calculated via

$$d^*({s_{ij}^*}^A, {s_{ij}^*}^B) = |{s_{ij}^*}^A - {s_{ij}^*}^B|$$

This procedure is repeated at least $P = 1000$ times, resulting in a vector of P test statistics d^* for each variable. Finally, a p-value for testing the null hypothesis that species i is equally associated in both networks is calculated as the proportion of distances based on the permuted data that are at least as large as the distance belonging to the observed data:

$$p = \frac{\sum \mathbb{1}\{D(s_{ij}^{*A}, s_{ij}^{*B}) \geq D(s_{ij}^A, s_{ij}^B)\}}{P}.$$

Since several hypotheses are tested simultaneously, multiple testing correction is needed for these nonparametric tests as well. The same adjustment methods as for the parametric tests are available in `NetCoMi`.

P-values arising from permutation tests are problematic because they may be exactly zero and a p-value equal to zero remains significant after adjustment for multiple testing. Furthermore, these p-values usually lead to a positively biased type I error rate (Phipson and Smyth, 2010). To overcome this issue, we use the approach proposed by Phipson and Smyth (2010) to estimate exact p-values, which is implemented in R via the `permp()` function (`statmod` package).

Gill et al. (2010) examined the goodness of their approach via a simulation study for a selection of connectivity scores. In that study, correlations, partial correlations as well as connectivity scores based on partial least squares are suggested as appropriate connectivity measures, for which their approaches are supposed to "work well in most circumstances"(Gill et al., 2010). Due to these results, we implemented the test procedure for all association measures (for measuring associations between taxa) available in `NetCoMi`.

The question might arise whether Fisher's z-values could be used as the basis for these tests instead of correlations. This has been done, for instance, in (McKenzie et al., 2016) for conducting differential correlation analysis. In Pesarin (2001) is stated that the power of permutation tests can be improved if based on an – at least approximately – symmetric data distribution. Since Fisher's z-values are approximately normal, permutation tests based on the z-transformed correlations may have a higher power to identify significantly differential correlated taxa. Thus, if a correlation coefficient is used as the association measure in `NetCoMi`, Fisher's z-values can also be used as the basis for the permutation tests instead of the estimated correlations themselves.

4.3 Discordant method

The "Discordant method" (Siska et al., 2016), for which also an `R` package named `discordant` (Siska and Kechris, 2019) exists, is based on mixture models instead of hypothesis testing and is illustrated in Figure S3. Since this approach is based on Fisher's z-transformation, it is only applicable to correlations.

4.3.1 Theoretical Background

Starting from the two correlation matrices belonging to group 1 and group 2, the goal is to assign each pair of the corresponding correlations r_1 and r_2 to one of the nine classes shown in Figure S3A. This 3×3 matrix describes all possible correlation scenarios between the groups that are considered by a mixture model. Note that this representation of the matrix is analog to the R code used in the `discordant` package (Siska and Kechris, 2019) (within the illustration of the algorithm pipeline shown in Siska et al. (2016) the groups are interchanged).

The approach is as follows: First, the sample correlations are transformed into Fisher's z-values via $z = \frac{1}{2}\log\frac{1+r}{1-r}$. A mixture model is then created based on these values, which is illustrated in Figure S3B. For each group, the Fisher z-values belong to one of the classes: 0, -, + (denominating the three possibilities: no correlation, negative, or positive correlation between two variables). The z-values within a class are assumed to be normally distributed with mean μ and variance σ^2 in group 1 and mean η and variance τ^2 in group 2. More precisely, correlations belonging to class "0" are distributed around 0, those in class "-" around an unknown negative mean μ_- and in class "+" around an unknown positive mean μ_+. For a data set with n variables (or taxa), there are also n variable pairs $k = (z_1, z_2)$ and since the classes (0,+,-) are assigned to single z-values, the paired values belong to one of the classes 1 to 9 of the class matrix in Figure S3A.

If the parameters were known, the class memberships could be easily computed based on the posterior probability that a variable pair belongs to a certain class. On the other hand, if the posteriors were known, a variable pair could be assigned to the class with the highest probability and the parameters would be determined via maximum likelihood estimation.

To solve this issue, an expectation-maximization (EM) algorithm is used, where the two steps are executed iteratively by fixing one component (posterior, or the parameters) and solving for the other.

The density of a pair of z-values is needed for the algorithm, which is defined as follows: Given that z_1 belongs to class i and z_2 belongs to class j where i and j can take the values 0 (class 0), 1 (class –), or 2 (class +), respectively, the density for the pair of z-values is expressed by:

$$f(z_1, z_2) = \sum_{i=0}^{2}\sum_{j=0}^{2}(\pi_{ij}\ \phi_{\mu_i,\sigma_i^2}(z_1)\ \phi_{\eta_j,\tau_j^2}(z_2))^{\mathbb{1}\{w_{ij}=1\}}.$$

Therein, ϕ_{μ_i,σ_i^2} and $\phi_{\eta_j,\tau_j^2}(z_2)$ denote the normal probability distribution functions for group 1 and group 2, respectively, and π_{ij} is the proportion of variables with z_1 belonging to class i and z_2 belonging to class j. The membership of the classes 1 to 9 representing all possible correlation scenarios is represented by w_{ij}. For example, if a variable pair belongs to class 7, then $w_{2\ 0} = 1$ and $w_{ij} = 0$ for $i \neq 2, j \neq 0$. The corresponding likelihood is defined as

$$L(\theta) = \prod_{p=1}^{n} f(z_1^p, z_2^p),$$

with θ denoting the set of parameters $\{\pi_{ij}, \mu_i, \sigma_i, \eta_j, \tau_j,\}$ for $i, j = 0, 1, 2$ (Siska et al., 2016).

The EM-algorithm is then as follows. First, an initial class membership is needed to determine the means and variances of the classes in both groups. Within the R function, the empirical standard deviations of z-values s_1 and s_2 are calculated in each group for this purpose. Based on these quantities, all variables with $-s_1 \leq z_1 \leq s_1$ or $s_2 \leq z_2 \leq s_2$, respectively, are assigned to class "0", those with a z-value smaller than the standard deviation to class "-" and those with a larger z-value to class "+". Means and variances are calculated for the resulting classes and used as initial parameters for the algorithm.

In the E-step, for each variable pair k, the posterior probabilities are determined for all nine classes (expressed by i and j, respectively) via

$$q_{ij}^r(k) = \mathbb{P}(w_{ij}(k) = 1\ |\theta^{r-1}, z_1, z_2),$$

where r is the current iteration number and θ^{r-1} the parameters of the mixture model resulting from the previous iteration step. q_{ij}^r is the updated posterior probability that a variable pair k belongs to class (i, j) in iteration r. Based on these probabilities, the parameters are updated within the M-step via maximum likelihood estimation. The corresponding formulas for re-estimating the parameters can be found in the supplementary material of Siska et al. (2016). These two steps are repeated until convergence of the algorithm leading to posterior probabilities $q_{ij}^r(k)$.

The term of interest, however, is not the probability for one of the nine classes, but the probability that a pair of taxa k is differentially correlated between the classes:

$$p(DC_k) = \sum_{i \neq j} q_{ij}^r(k),$$

which is the sum of posterior probabilities for the off-diagonal classes in the class matrix (Figure S3A). The authors do not suggest a certain threshold above which the taxa are considered as differentially correlated. In `NetCoMi`, we use a default value of 0.8, so that taxa are identified as differentially correlated if the sum of probabilities for the off-diagonal classes is greater than or equal to 0.8.

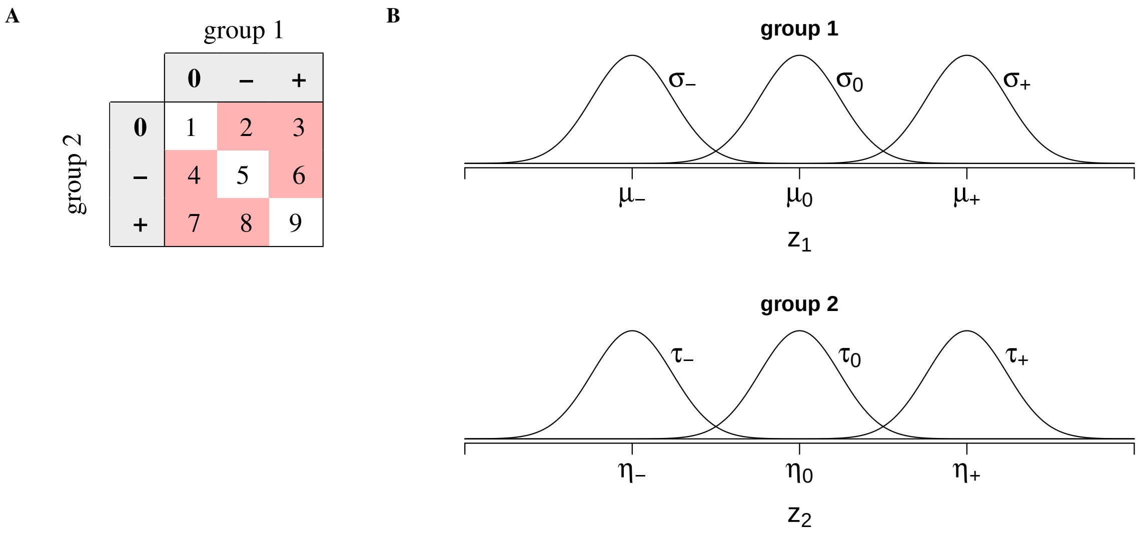

Figure S3: Class matrix and mixture model explaining the Discordant method, which is based on parts of the Discordant algorithm pipeline published in Siska et al. (2016). **A** Class matrix describing all possible correlation scenarios considered in the Discordant approach. Group 1 and group 2 are the two subsets of interest into which the count matrix has been divided. Class 4 means, for example, that two variables are not correlated in group 1 and negatively correlated in group 2. Variable pairs belonging to the off-diagonal classes (colored in red) are considered as differentially correlated. **B** Mixture model with three classes: 0, – and +, for group 1 and group 2 respectively. The z-values within a class are assumed to be normally distributed with mean μ and variance σ^2 in group 1 and mean η and variance τ^2 in group 2.

4.3.2 Comparison to other methods

Siska et al. (2016) compared their approach to existing methods via a simulation study and additionally by applying the methods to real data for evaluating the performance of the Discordant method. The compared methods for differential correlation analysis were Fisher's z-test, a linear interaction model, and "EBcoexpress" (Dawson et al., 2012) (a bivariate mixture model based on a hierarchical model). P-values were returned by the former and posterior probabilities by EBcoexpress and Discordant, which are not directly comparable. Thus, the variable pairs were ranked by increasing p-values (Fisher's z, EBcoexpress) or decreasing posterior (Discordant). Then, the rankings were compared regarding statistical significance between the methods. Pearson's correlation coefficient was used as correlation measure in each case.

The study concluded that specificity is the same for all methods but Discordant distinguishes from the alternative methods by a higher sensitivity. Thus, more truly differential correlated variable pairs are identified via the Discordant method. Furthermore, the ability of a procedure to predict the paired correlation scenarios expressed by the class matrix were investigated (Siska et al., 2016). It has been shown that all methods have similar performance in identifying the extreme classes where the variables are positively correlated in one group and negative in the other. However, the Discordant method has greater power for detecting differentially correlated variable pairs if the correlation is absent in one of the groups. (Siska et al., 2016).

5 Case study: Applying NetCoMi to real data from GABRIELA study

5.1 Data preprocessing

Table S4: After denoising the data with QIIME-2 using DADA-2, these preprocessing steps where performed for the mattress dust and nasal swabs data, respectively.

Preprocessing step	Total number of taxa	Total number of samples
Mattress dust:		
Original data on OTU level	61666	1044
Remove uncharacterized phyla	59503	1044
Keep only bacteria	59143	1044
Remove Chloroflexi sequences	56784	1044
Remove Chloroplast sequences	54260	1044
Remove mitochondrial sequences	50533	1044
Remove NAs	45813	1044
Assign higher taxonomic rank to uncultured bacteria	45813	1044
Merge down to genus level	2894	1044
Remove samples with less than 1000 reads in total	2894	1022
Remove taxa with less than 1000 reads in total	707	1022
Nasal swabs:		
Original data on OTU level	46675	1127
Remove uncharacterized phyla	27995	1127
Keep only bacteria	27524	1127
Remove Chloroflexi sequences	26935	1127
Remove Chloroplast sequences	25462	1127
Remove mitochondrial sequences	23937	1127
Remove NAs	20073	1127
Assign higher taxonomic rank to uncultured bacteria	20073	1127
Merge down to genus level	1680	1127
Remove samples with less than 1000 reads in total	1680	1033
Remove taxa with less than 1000 reads in total	467	1033

5.2 Network properties of the single microbial association network

Table S5: Properties of the network in Figure 4 in the main text. Shown are: a) global network properties, b) frequency table of the clusters, c) the five detected hub nodes, d)-g) centrality values of the ten genera with the highest centrality in decreasing order.

a) Global properties

average path length	1.58208
clustering coefficient	0.28367
modularity	0.44044
edge density	0.10586
vertex connectivity	2
edge connectivity	2

b) Clusters

Name	Frequency
1	23
2	31
3	17
4	29

c) Hub taxa

[Eubacterium] coprostanoligenes group
Aerococcus
Atopostipes
Brachybacterium
Rikenellaceae RC9 gut group

d) Degree

**Genus**	**Centrality**
[Eubacterium] coprostanoligenes group	0.22222
Atopostipes	0.19192
Brachybacterium	0.18182
Dialister	0.17172
Sphingomonas	0.16162
Porphyromonas	0.16162
Acidiphilium	0.16162
Aerococcus	0.16162
Glutamicibacter	0.16162
Rikenellaceae RC9 gut group	0.16162

e) Betweenness centrality

**Genus**	**Centrality**
Porphyromonas	0.05607
[Eubacterium] coprostanoligenes group	0.05236
Leuconostoc	0.04226
Brachybacterium	0.04061
Sphingomonas	0.03772
Clostridium sensu stricto 1	0.03772
Dialister	0.03525
Acidiphilium	0.03504
Blautia	0.03443
Pseudomonas	0.03175

f) Closeness centrality

**Genus**	**Centrality**
[Eubacterium] coprostanoligenes group	0.87739
Atopostipes	0.83849
Brachybacterium	0.83272
Porphyromonas	0.81466
Rikenellaceae RC9 gut group	0.81272
Glutamicibacter	0.80265
Clostridium sensu stricto 1	0.80171
Dialister	0.80013
Kocuria	0.79544
Prevotella 9	0.79531

g) Eigenvector centrality

**Genus**	**Centrality**
Atopostipes	1.00000
[Eubacterium] coprostanoligenes group	0.89872
Brachybacterium	0.89250
Rikenellaceae RC9 gut group	0.83003
Aerococcus	0.79830
Glutamicibacter	0.77511
Romboutsia	0.74624
Psychrobacter	0.70911
Jeotgalicoccus	0.70841
Prevotella 9	0.69081

5.3 Microbial association network with "unsigned" distance

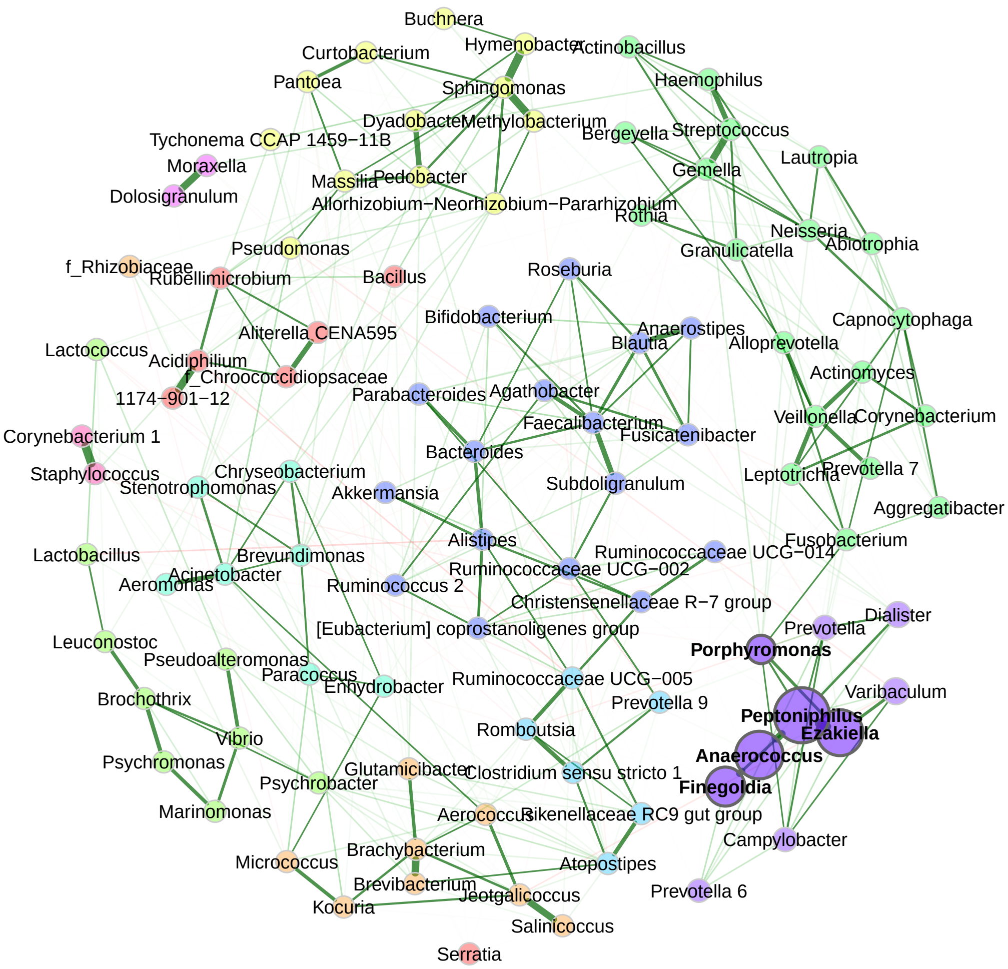

Figure S4: Bacterial associations for the combined data set with samples from Ulm and Munich (with 100 taxa and 1022 samples). The SPRING method Yoon et al. (2019) is used as an association measure. The estimated partial correlations are transformed to dissimilarities via the "unsigned" distance metric and the corresponding similarities are used as edge weights. Eigenvector centrality is used for defining hubs (nodes with a centrality value above the empirical 95% quantile) and scaling node sizes. Hubs are highlighted by bold text and borders. Node colors represent clusters, which are determined using greedy modularity optimization. Green edges correspond to positive correlations and red edges to negative ones.

Table S6: Properties of the network in Figure S4 with “unsigned” distance. Shown are: a) global network properties, b) frequency table of the clusters, c) the five detected hub nodes, d)-g) centrality values of the ten genera with the highest centrality in decreasing order.

a) Global properties

average path length	2.34052
clustering coefficient	0.28367
modularity	0.38863
edge density	0.10586
vertex connectivity	2
edge connectivity	2

b) Clusters

Name	Frequency
1	7
2	9
3	12
4	9
5	19
6	7
7	6
8	17
9	10
10	2
11	2

c) Hubs

Anaerococcus
Ezakiella
Finegoldia
Peptoniphilus
Porphyromonas

d) Degree

**Genus**	**Centrality**
[Eubacterium] coprostanoligenes group	0.22222
Atopostipes	0.19192
Brachybacterium	0.18182
Dialister	0.17172
Sphingomonas	0.16162
Porphyromonas	0.16162
Acidiphilium	0.16162
Aerococcus	0.16162
Glutamicibacter	0.16162
Rikenellaceae RC9 gut group	0.16162

e) Betweenness centrality

**Genus**	**Centrality**
Porphyromonas	0.05607
[Eubacterium] coprostanoligenes group	0.05483
Leuconostoc	0.04082
Brachybacterium	0.03979
Sphingomonas	0.03793
Clostridium sensu stricto 1	0.03772
Acidiphilium	0.03711
Blautia	0.03649
Pseudomonas	0.03546
Dialister	0.03381

f) Closeness centrality

**Genus**	**Centrality**
[Eubacterium] coprostanoligenes group	0.60208
Atopostipes	0.57105
Brachybacterium	0.56016
Rikenellaceae RC9 gut group	0.55321
Porphyromonas	0.55316
Glutamicibacter	0.54612
Dialister	0.54492
Prevotella 9	0.54201
Prevotella	0.54125
Kocuria	0.54088

g) Eigenvector centrality

**Genus**	**Centrality**
Peptoniphilus	1.00000
Anaerococcus	0.77458
Ezakiella	0.72303
Finegoldia	0.51386
Porphyromonas	0.19426
Varibaculum	0.14364
Dialister	0.11871
Prevotella	0.08406
Campylobacter	0.06359
Prevotella 6	0.03558

5.4 Additional results for network comparison using SPRING as association measure

Table S7: Properties of the networks constructed for the two study centers Munich and Ulm shown in Figure 4 in the main text. Shown are: a) Frequency table of clusters in the Munich network. b) Frequency table of clusters in the Ulm network. c) Detected hub nodes in both groups. d)-g) Centrality values of the genera with the highest centrality in decreasing order. The upper part of the table contains the five genera with the highest centrality in Munich and the lower part those with the highest centrality in Ulm, respectively. Thus, a genus can occur twice in the same table.

a) Clusters at Munich

Name	Frequency
1	29
2	6
3	19
4	16
5	30

b) Clusters at Ulm

Name	Frequency
1	36
2	16
3	17
4	29
5	2

c) Hubs nodes

Munich	Ulm
[Eubacterium] coprostanoligenes group	[Eubacterium] coprostanoligenes group
Aerococcus	Atopostipes
Atopostipes	Brachybacterium
Brachybacterium	Pedobacter
Pseudomonas	Rikenellaceae RC9 gut group

d) Degree

Genus	Munich	Ulm
**Highest values in the Munich group:**		
Pseudomonas	0.20202	0.13131
Atopostipes	0.19192	0.18182
Aerococcus	0.18182	0.12121
[Eubacterium] coprostanoligenes group	0.18182	0.19192
Brachybacterium	0.17172	0.16162
**Highest values in the Ulm group:**		
[Eubacterium] coprostanoligenes group	0.18182	0.19192
Rikenellaceae RC9 gut group	0.10101	0.19192
Atopostipes	0.19192	0.18182
Pedobacter	0.10101	0.17172
Neisseria	0.13131	0.16162

e) Betweenness centrality

Genus	Munich	Ulm
**Highest values in the Munich group:**		
Clostridium sensu stricto 1	0.04968	0.02494
Brachybacterium	0.04205	0.0235
Aerococcus	0.03628	0.01134
Dialister	0.03546	0.01917
Corynebacterium 1	0.03484	0.01216
**Highest values in the Ulm group:**		
Porphyromonas	0.02123	0.04473
Sphingomonas	0.0334	0.03999
[Eubacterium] coprostanoligenes group	0.03072	0.03669
Gemella	0.01587	0.03608
Neisseria	0.02597	0.03546

f) Closeness centrality

Genus	Munich	Ulm
**Highest values in the Munich group:**		
Pseudomonas	0.84238	0.75576
Aerococcus	0.83892	0.77209
Atopostipes	0.83707	0.82352
Brachybacterium	0.83634	0.79409
[Eubacterium] coprostanoligenes group	0.83111	0.83719
**Highest values in the Ulm group:**		
Rikenellaceae RC9 gut group	0.73494	0.84813
[Eubacterium] coprostanoligenes group	0.83111	0.83719
Atopostipes	0.83707	0.82352
Ruminococcaceae UCG-005	0.797	0.82
Porphyromonas	0.72098	0.80468

g) Eigenvector centrality

Genus	Munich	Ulm
**Highest values in the Munich group:**		
Atopostipes	1	0.9732
Brachybacterium	0.90898	0.86592
[Eubacterium] coprostanoligenes group	0.89306	0.81747
Aerococcus	0.87499	0.70126
Pseudomonas	0.85217	0.41707
**Highest values in the Ulm group:**		
Rikenellaceae RC9 gut group	0.59459	1
Atopostipes	1	0.9732
Pedobacter	0.5264	0.87283
Brachybacterium	0.90898	0.86592
[Eubacterium] coprostanoligenes group	0.89306	0.81747

Table S8: Results from testing centralities of the networks in Figure 5 in the main text for group differences (via permutation tests using 1000 permutations). Shown are respectively the computed measure for Munich and Ulm, the absolute difference, and the p-value for testing the null hypothesis $H_0 : |\text{diff}| = 0$. P-values are adjusted for multiple testing using the adaptive Benjamini-Hochberg method Benjamini and Hochberg (2000), where the proportion of true H_0 is determined according to Langaas et al. Langaas et al. (2005). For all four measures, the values are normalized to [0,1] as described in Table 2 in the main text.

Genus	Munich	Ulm	abs. difference	adj. p-value
Degree (weighted):				
Corynebacterium 1	0.162	0.071	0.091	0.199046
Rikenellaceae RC9 gut group	0.101	0.192	0.091	0.829358
Lactobacillus	0.121	0.051	0.071	0.829358
Pedobacter	0.101	0.172	0.071	0.829358
Pseudomonas	0.202	0.131	0.071	0.996225
Ruminococcaceae UCG-002	0.162	0.101	0.061	0.829358
Aerococcus	0.182	0.121	0.061	0.996225
Tychonema CCAP 1459-11B	0.071	0.121	0.051	0.996225
Blautia	0.152	0.101	0.051	0.996225
Agathobacter	0.091	0.040	0.051	0.829358
Betweenness centrality:				
Aerococcus	0.036	0.011	0.025	0.978332
Clostridium sensu stricto 1	0.050	0.025	0.025	0.978332
Porphyromonas	0.021	0.045	0.024	0.978332
Corynebacterium 1	0.035	0.012	0.023	0.978332
Lactobacillus	0.026	0.003	0.022	0.978332
Glutamicibacter	0.025	0.004	0.021	0.978332
Gemella	0.016	0.036	0.020	0.978332
Brachybacterium	0.042	0.024	0.019	0.978332
Haemophilus	0.031	0.013	0.017	0.978332
Dialister	0.035	0.019	0.016	0.978332
Closeness centrality:				
Corynebacterium 1	0.825	0.690	0.135	0.884116
Psychromonas	0.472	0.604	0.131	0.884116
Rikenellaceae RC9 gut group	0.735	0.848	0.113	0.884116
Finegoldia	0.694	0.591	0.103	0.884116
Tychonema CCAP 1459-11B	0.646	0.739	0.093	0.884116
Chroococcidiopsaceae	0.628	0.721	0.093	0.884116
Alloprevotella	0.657	0.749	0.092	0.884116
Brochothrix	0.574	0.665	0.091	0.884116
Paracoccus	0.646	0.734	0.088	0.884116
Pseudomonas	0.842	0.756	0.087	0.884116
Eigenvector centrality:				
Pseudomonas	0.852	0.417	0.435	0.574582
Rikenellaceae RC9 gut group	0.595	1.000	0.405	0.574582
Ruminococcaceae UCG-002	0.767	0.379	0.389	0.574582
Corynebacterium 1	0.670	0.290	0.379	0.574582
Pedobacter	0.526	0.873	0.346	0.833965
Blautia	0.710	0.368	0.342	0.833965
Tychonema CCAP 1459-11B	0.226	0.558	0.332	0.574582
Ruminococcus 2	0.694	0.370	0.325	0.833965
Agathobacter	0.452	0.157	0.295	0.574582
Rhizobiaceae	0.324	0.602	0.278	0.833965

Significance codes: ***: 0.001, **: 0.01, *: 0.05, .: 0.1

Table S9: Abbreviations of node labels used in Figure 5 in the main text.

**Renamed labels**	**Original labels**
Streptoco	Streptococcus
Staphyloc	Staphylococcus
Acinetoba	Acinetobacter
Haemophil	Haemophilus
Coryne’1	Corynebacterium 1
Sphingomo	Sphingomonas
Bacteroid	Bacteroides
Paracoccu	Paracoccus
Pseudomon	Pseudomonas
Anaerococ	Anaerococcus
Porphyrom	Porphyromonas
Faecaliba	Faecalibacterium
Prevot’9	Prevotella 9
Peptoniph	Peptoniphilus
Lactobaci	Lactobacillus
Prevot	Prevotella
Neisseria	Neisseria
Ezakiella	Ezakiella
Enhydroba	Enhydrobacter
Moraxella	Moraxella
Gemella	Gemella
Rothia	Rothia
Actinobac	Actinobacillus
Lactococc	Lactococcus
Micrococc	Micrococcus
Alloprevo	Alloprevotella
Veillonel	Veillonella
Kocuria	Kocuria
Methyloba	Methylobacterium
Agathobac	Agathobacter
Massilia	Massilia
Finegoldi	Finegoldia
Pedobacte	Pedobacter
Actinomyc	Actinomyces
Hymenobac	Hymenobacter
Pantoea	Pantoea
Subdoligr	Subdoligranulum
Bifidobac	Bifidobacterium
Psychroba	Psychrobacter
Allorhizo	Allorhizobium-Neorhizobium-Pararhizobium-Rhizobium
Chryseoba	Chryseobacterium
Blautia	Blautia
Fusobacte	Fusobacterium
Jeotgalic	Jeotgalicoccus
Marinomon	Marinomonas
Coryne	Corynebacterium
Prevot’7	Prevotella 7
Brochothr	Brochothrix
Rumino’us	Ruminococcus 2
Brevundim	Brevundimonas

**Renamed labels**	**Original labels**
Serratia	Serratia
Leptotric	Leptotrichia
Brachybac	Brachybacterium
Brevibact	Brevibacterium
Psychromo	Psychromonas
Clostridi	Clostridium sensu stricto 1
Rumino’ace	Ruminococcaceae UCG-005
Pseudoalt	Pseudoalteromonas
Prevot’6	Prevotella 6
Granulica	Granulicatella
Chrooco(F)	Chroococcidiopsaceae(F)
Alistipes	Alistipes
Varibacul	Varibaculum
Bergeyell	Bergeyella
Acidiphil	Acidiphilium
Romboutsi	Romboutsia
Campyloba	Campylobacter
1174-901-	1174-901-12
Aerococcu	Aerococcus
Dolosigra	Dolosigranulum
Rhizobi(F)	Rhizobiaceae(F)
Leuconost	Leuconostoc
Vibrio	Vibrio
Lautropia	Lautropia
Dialister	Dialister
[Eubacter]	[Eubacterium] coprostanoligenes group
Stenotrop	Stenotrophomonas
Roseburia	Roseburia
Aggregati	Aggregatibacter
Bacillus	Bacillus
Christens	Christensenellaceae R-7 group
Parabacte	Parabacteroides
Rumino’ace	Ruminococcaceae UCG-014
Rubellimi	Rubellimicrobium
Curtobact	Curtobacterium
Atopostip	Atopostipes
Glutamici	Glutamicibacter
Akkermans	Akkermansia
Abiotroph	Abiotrophia
Buchnera	Buchnera
Rumino’ace	Ruminococcaceae UCG-002
Aliterell	Aliterella CENA595
Rikenella	Rikenellaceae RC9 gut group
Anaerosti	Anaerostipes
Tychonema	Tychonema CCAP 1459-11B
Capnocyto	Capnocytophaga
Dyadobact	Dyadobacter
Aeromonas	Aeromonas
Salinicoc	Salinicoccus
Fusicaten	Fusicatenibacter

5.5 Differential network

A *differential network*, where edges exist between differentially associated taxa, is constructed by passing the object returned from `netConstruct()` to `diffnet()`. We use the permutation procedure (see Table 5 in the main text) to test the associations estimated via the SPRING method (Yoon et al., 2019) for differences. For our data, none of the correlations was significantly different between Ulm and Munich after multiple testing correction (by controlling the local false discovery rate (Efron, 2005)) so that no edges exist in the differential network. Note that this might be due to the fact that permutation p-values have a lower limit, which is related to the number of permutations (in our example 0.001 because of 1000 permutations). Thus, a p-value cannot be arbitrarily small, even if for a pair of taxa the observed difference is much higher than the differences arising from the permuted data.

5.6 Comparison of sample similarity networks

Table S10: Properties of the networks constructed for mattress dust and nasal swabs shown in Figure 6 in the main text. Shown are: a) Frequency table of clusters in the Mattress network. b) Frequency table of clusters in the Nose network. c) Detected hub nodes in both groups. d)-g) Centrality values of the genera with the highest centrality in decreasing order. The upper part of the table contains the five genera with the highest centrality in the mattress network and the lower part those with the highest the centrality in the nose network, respectively. Thus, a genus can occur twice in the same table.

a) Clusters in the mattress network

Name	Frequency
1	560
2	221
3	198

b) Clusters in the nose network

Name	Frequency
1	419
2	245
3	315

c) Hub nodes (subjects)

Mattress	Nose
1054	1069
1174	1082
1307	1271
1392	1360
1434	1454
1510	1687
1581	1689
1793	1777
1896	1933
2053	2048

d) Degree

**Subject**	**Mattress**	**Nose**
**Highest values in the Mattress group:**		
1174	0.20859	0.00307
1434	0.16053	0.00307
1307	0.15644	0.00307
1510	0.15542	0.00511
1896	0.14008	0.00307
**Highest values in the Nose group:**		
1069	0.00307	0.19632
2048	0.00409	0.16462
1268	0.00307	0.10327
1360	0.00307	0.08998
1787	0.00307	0.08998

e) Betweenness centrality

**Subject**	**Mattress**	**Nose**
**Highest values in the Mattress group:**		
1174	0.31951	0.00001
1896	0.15342	0.00013
2053	0.14126	0
1510	0.13824	0.00012
1434	0.13787	0
**Highest values in the Nose group:**		
2048	0	0.45619
1069	0	0.32614
1787	0	0.17234
1249	0.00417	0.11410
1422	0	0.10124

f) Closeness centrality

**Subject**	**Mattress**	**Nose**
**Highest values in the Mattress group:**		
1174	4.27319	1.05886
1750	4.15324	1.46256
1203	3.90505	1.42563
1510	3.90289	1.20292
1434	3.87384	1.38911
**Highest values in the Nose group:**		
1069	2.01941	2.28632
2048	2.10909	2.28023
1454	2.67159	2.08319
1777	2.25869	2.02704
1787	1.94227	2.02443

g) Eigenvector centrality

**Subject**	**Mattress**	**Nose**
**Highest values in the Mattress group:**		
1174	1	0.00151
1434	0.89765	0.03591
1510	0.863	0.00078
1307	0.72712	0.0005
1896	0.70185	0.00122
**Highest values in the Nose group:**		
1069	0.048	1
1360	0.04889	0.59326
2048	0.03478	0.40901
1454	0.08403	0.38686
1777	0.0695	0.30589

Table S11, S12, and S13 contain results of the network comparison of the subject-to-subject-networks between mattress dust and nasal swabs shown in Figure 6 in the main text. The determined adjusted Rand index is -0.003 with a p-value of 0.382 meaning that the clusterings are not significantly different from two random clusterings.

Table S11: Jaccard index values corresponding to Figure 6. Index values j express the similarity of the sets of most central nodes and also of the sets of hub taxa between the two networks. "Most central" nodes are those with a centrality value above the empirical 75% quantile. Jaccard's index is 0 if the sets are completely different and 1 for exactly equal sets. $P(J \leq j)$ is the probability that Jaccard's index takes for the present total number of taxa a value less than or equal to the calculated index j ($P(J \geq j)$ is defined analogously). Here, the index is significantly close to zero for all measures, meaning that the sets of most central are considerably different between the groups. The hubs are completely different leading to a Jaccard index of 0.

	j	P($J \leq j$)	P($J \geq j$)
degree	0.111	0.000000 ***	1
betweenness centr.	0.140	0.000000 ***	1
closeness centr.	0.132	0.000000 ***	1
eigenvec. centr.	0.153	0.000000 ***	1
hub taxa	0.000	0.000301 ***	1
Significance codes:	***: 0.001, **: 0.01, *: 0.05, .: 0.1		

Table S12: Results from testing global network metrics of the networks in Figure 6 for group differences (via a permutation procedure using 1000 permutations). Shown are respectively the computed measure for Mattress and Nose, the absolute difference, and the p-value for testing the null hypothesis $H_0 : |\text{diff}| = 0$.

	Mattress	Nose	abs. difference	p-value
Global network measures:				
average path length	0.516	0.892	0.375	0.000999 ***
clustering coefficient	0.029	0.055	0.026	0.004995 ***
modularity	0.396	0.529	0.132	0.011988 ***
edge density	0.006	0.006	0.000	0.864136
Significance codes:	***: 0.001, **: 0.01, *: 0.05, .: 0.1			

Table S13: Results from testing centrality measures of the networks in Figure 6 for group differences (via a permutation procedure using 1000 permutations). Shown are respectively the computed measure for Mattress and Nose, the absolute difference, and the p-value for testing the null hypothesis $H_0 : |\text{diff}| = 0$. The p-values are adjusted for multiple testing using the adaptive Benjamini-Hochberg method (Benjamini and Hochberg, 2000), where the proportion of true H_0 is determined according to Langaas et al. (Langaas et al., 2005). These results contain 10 subjects with the highest absolute group difference. All measures are normalized to [0,1].

Subject	Mattress	Nose	abs. difference	adj. p-value
Degree (weighted):				
1174	0.209	0.003	0.206	0.017504 *
1069	0.003	0.196	0.193	0.017504 *
2048	0.004	0.165	0.161	0.017504 *
1434	0.161	0.003	0.157	0.017504 *
1307	0.156	0.003	0.153	0.039188 *
1510	0.155	0.005	0.150	0.656391
1896	0.140	0.003	0.137	0.069617 .
2053	0.140	0.003	0.137	0.355379
1793	0.122	0.003	0.119	0.317705
1268	0.003	0.103	0.100	0.017504 *
Betweenness centrality:				
2048	0.000	0.456	0.456	0.018325 *
1069	0.000	0.326	0.326	0.018325 *
1174	0.320	0.000	0.320	0.029196 *
1787	0.000	0.172	0.172	0.018325 *
1896	0.153	0.000	0.153	0.381763
2053	0.141	0.000	0.141	0.880649
1510	0.138	0.000	0.138	0.880649
1434	0.138	0.000	0.138	0.207465
1581	0.131	0.000	0.131	0.018325 *
1307	0.126	0.000	0.126	0.189892
Closeness centrality:				
1174	4.273	1.059	3.214	0.002017 **
1392	3.843	0.967	2.876	0.001345 **
1084	3.783	1.070	2.714	0.004882 **
1510	3.903	1.203	2.700	0.010759 *
1750	4.153	1.463	2.691	0.010289 *
1896	3.816	1.145	2.671	0.005505 **
1793	3.811	1.219	2.592	0.006678 **
1307	3.728	1.144	2.584	0.004184 **
1148	3.503	0.952	2.551	0.001345 **
1434	3.874	1.389	2.485	0.004184 **
Eigenvector centrality:				
1174	1.000	0.002	0.998	0.048389 *
1069	0.048	1.000	0.952	0.048389 *
1510	0.863	0.001	0.862	0.338618
1434	0.898	0.036	0.862	0.119550
1307	0.727	0.000	0.727	0.154056
1896	0.702	0.001	0.701	0.174201
1360	0.049	0.593	0.544	0.159400
1793	0.538	0.003	0.535	0.239424
1054	0.482	0.008	0.474	0.082953 .
2053	0.520	0.076	0.444	0.287656

Significance codes: ***: 0.001, **: 0.01, *: 0.05, .: 0.1

6 Integrating external networks into NetCoMi

Here, we demonstrate how an existing network is integrated into `NetCoMi`'s workflow. The networks in our example have been constructed using the BAnOCC method (Schwager et al., 2017) for association estimation. BAnOCC uses a Bayesian approach to infer associations from compositional data.

Using `NetCoMi`, we analyze the two non-trivial correlation networks shown in Figure 5 in the BAnOCC paper (Schwager et al., 2017): Stool and Buccal mucosa. Therein, only genera with a significant association above a threshold of 0.15 are connected. The estimated posterior median and 95% interval for each connected node pair is provided in Table S1 (Stool network) and Table S2 (Buccal network) in (Schwager et al., 2017).

We use the posterior mean as edge weight to construct edge lists for both networks. Using `igraph`'s function `as_adjacency_matrix()`, the edge lists are transformed into weighted adjacency matrices, which are passed to `NetCoMi`'s function `netConstruct()`. Note that Table S1 and S2 in (Schwager et al., 2017) contain a feature "Viruses_noname", which is not existent in Figure 5 in the BAnOCC paper (Schwager et al., 2017). In line with the original network plots, we exclude the "Viruses_noname" feature from the edge lists.

`netConstruct()` returns a `microNet` object, which is then passed to `netAnalyze()` to compute the network properties shown in Table S14. The network plots are displayed in Figure S5. Compared to the original network plots, the network clusters are clearly visible in Figure S5 due to a different node placement. `NetCoMi`'s network plots furthermore display the computed local network properties (node centrality, hub nodes, and cluster assignment), which enables a visual network analysis.

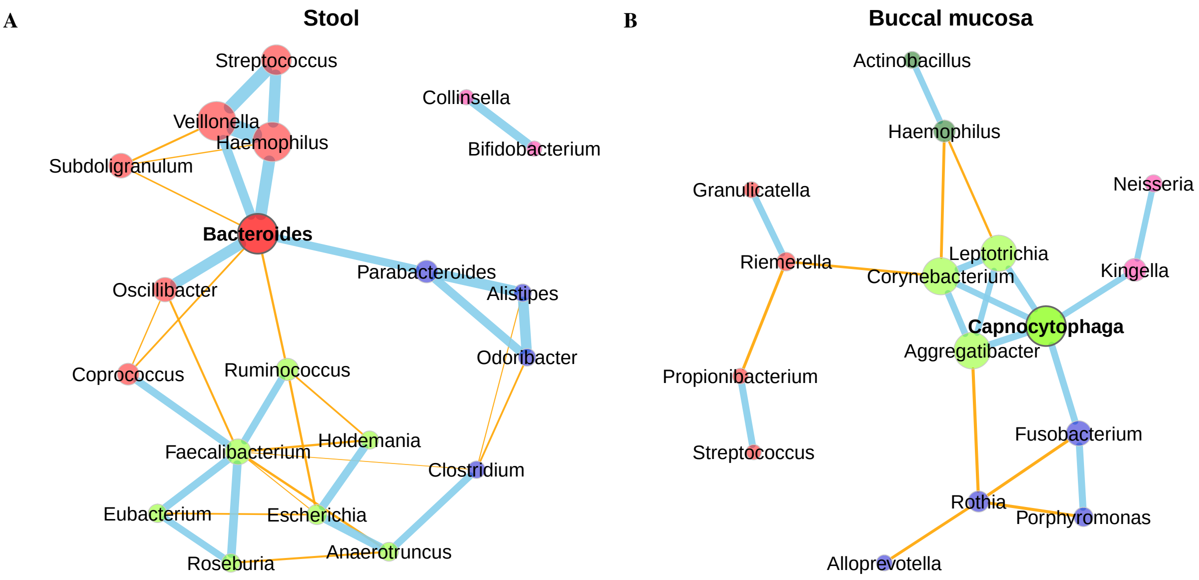

Figure S5: Stool and buccal mucosa networks shown in Figure 5 in the BAnOCC paper (Schwager et al., 2017), which are reconstructed and analyzed using NetCoMi's functions. In line with the original paper, blue edges correspond to positive associations and orange edges to negative ones. Node colors represent clusters determined via fast greedy modularity optimization. Node sizes are scaled according to eigenvector centrality. Hubs are nodes with an eigenvector centrality above the 95% quantile of the empirical centrality distribution. A force-directed layout is used for node placement.

Table S14: Network properties of the networks in Figure S5 computed with NetCoMi. Shown are: a) Frequency table of clusters determined for both networks. Clusters are identified using fast greedy modularity optimization. b) Hub nodes of both networks. Hubs are nodes with an eigenvector centrality above the 95% quantile of the empirical centrality distribution. c) Global network properties of both groups. d) and e) Centrality values for the stool and buccal mucosa network. For each centrality measure, the five genera with highest centrality are shown.

a) Clusters with node frequencies

Cluster	Stool	Buccal
1	7	4
2	4	4
3	7	4
4	2	2
5		2

b) Hub nodes

Stool	Buccal
Bacteroides	Capnocytophaga

c) Global properties

	Stool	Buccal
average path length	1.46914	1.88365
clustering coefficient	0.43200	0.36000
modularity	0.48418	0.43197
edge density	0.18947	0.17500
vertex connectivity	0	1
edge connectivity	0	1

d) Centrality values of stool network

Genus	Centrality
**Degree (weighted):**	
Faecalibacterium	0.47368
Bacteroides	0.36842
Escherichia	0.26316
Veillonella	0.21053
Clostridium	0.21053
**Betweenness:**	
Bacteroides	0.39181
Faecalibacterium	0.25146
Oscillibacter	0.13450
Parabacteroides	0.12281
Haemophilus	0.08187
**Closeness:**	
Bacteroides	0.92542
Faecalibacterium	0.91003
Haemophilus	0.79655
Veillonella	0.78021
Ruminococcus	0.76146
**Eigenvector:**	
Bacteroides	1.00000
Haemophilus	0.99417
Veillonella	0.98125
Streptococcus	0.64780
Faecalibacterium	0.48023

e) Centrality values of buccal mucosa network

Genus	Centrality
**Degree (weighted):**	
Corynebacterium	0.33333
Capnocytophaga	0.33333
Leptotrichia	0.26667
Rothia	0.26667
Aggregatibacter	0.26667
**Betweenness:**	
Corynebacterium	0.57143
Capnocytophaga	0.40952
Riemerella	0.36190
Aggregatibacter	0.20952
Rothia	0.14286
**Closeness:**	
Capnocytophaga	0.95285
Corynebacterium	0.92635
Aggregatibacter	0.86712
Leptotrichia	0.84970
Fusobacterium	0.77451
**Eigenvector:**	
Capnocytophaga	1.00000
Corynebacterium	0.89312
Aggregatibacter	0.87608
Leptotrichia	0.85623
Fusobacterium	0.40764

7 Comparison of network construction methods using GABRIELA data

We consider the following five cases to demonstrate the variability of networks constructed via different methods:

- **One of each** (one representative of each association type):
  - SparCC
  - Proportionality (Prop)
  - SPRING
- **Correlations** (compositionally aware correlation estimation methods):
  - Pearson correlation applied to clr transformed data (Pearson + clr)
  - SparCC
  - CCLasso
  - CCREPE
- **Conditional dependence** (compositionally aware estimators of conditional dependence):
  - SPRING
  - SpiecEasi with neighborhood selection of Meinshausen and Bühlmann (MB)
  - SpiecEasi with graphical lasso (glasso)
  - gCoda
- **Normalization** (Pearson correlation with four normalization methods and unit pseudo counts for zero replacement):
  - Total sum scaling (TSS)
  - Rarefying
  - Centered log-ratio transformation (CLR)
  - Variance stabilizing transformation (VST)
- **Zero replacement** (Pearson correlation in combination with four zero replacement methods and clr normalization):
  - Unit pseudo count (CLR from "Normalization" setting)
  - Multiplicative imputation (multImp)
  - Modified EM alr-algorithm (EMalr)
  - Bayesian-multiplicative treatment (bayesMult)

For this comparative study, we use mattress dust data from the GABRIELA study ($N = 1022, p = 707$). Like in the main text, we select the 100 most frequent genera, leading to a 1022×100 count matrix. The network plots are shown in Figures S6, S7, S8, and S9. Nodes are colored according to their taxonomy at phylum level in order to visualize how well the clusters represent bacterial phyla. Node sizes express the sum of relative frequencies (absolute counts divided by the total number of counts per sample) of a genus, which facilitates the visual network comparison.

Figures S10 and S11 show venn diagrams for the five aforementioned settings, together with a consensus network, where an edge exists between taxa that are connected in all networks of the respective setting. As before, colors represent phyla and node sizes relative frequencies. The plots reveal a high variability between the association estimation methods. As expected, there is a large overlap between correlation and proportionality (Figure S10A) and both association types lead to denser networks than the SPRING method. However, also within an association type (Figures S10C, S10D, S10E, and S10F) there are considerable differences between the approaches. We also see strong differences between the considered normalization methods (Figures S11A and S11B). This is in accordance with the results shown in (Badri et al., 2020), where the CLR and VST transformation lead to similar association estimates, which are strongly different from other (not compositionally aware) normalization methods. The most stable results are seen for different zero replacement methods (Figure S11C) so that the corresponding consensus network (Figure S11D) is also the densest one.

The computed global network properties describing the overall network structure are available in Table S15. Also these network characteristics differ strongly between the network construction methods. The multiple-site similarity measure proposed by Diserud and Ødegaard (2007) is used to express the similarity of centrality values within each setting (see Table S16). More precisely, for each centrality measure and each network a set of most central nodes is determined and the index indicates how similar the three or four sets of a setting are. The similarity index (equation 2.3 in (Diserud and Ødegaard, 2007)) ranges from 0 to 1, where one corresponds to exactly equal sets of most central nodes and zero means that the sets are completely different.

We consider two definitions of "most central nodes". Most central are nodes with a centrality value either above the 75% quantile or the 95% quantile of the empirical distribution of centralities, for each centrality measure respectively. Table S16 contains for both quantiles the computed similarity indices of all four centrality measures implemented in `NetCoMi` and all five settings considered in this section. Besides the similarity index, also the intersection of all sets of a setting are given in Table S16. These results are on the one hand important with regard to hub nodes as part of the network analysis workflow and on the other hand for calculating the Jaccard index, which expresses the similarity of most central nodes as part of `NetCoMi`'s network comparison workflow. To give an example, for the 95% quantile the intersection of most central nodes regarding eigenvector centrality is zero for all five settings (TableS16). Since we define hub nodes equally (as nodes with an eigenvector centrality value above the empirical 95% quantile), the results indicate that in our examples no hub node is existent in all networks of a setting.

To summarize, the comparative study shown in this section illustrates that different network construction methods might lead to considerable different networks and accordingly also to different local and global network properties, which must be considered when analyzing and comparing microbial association networks.

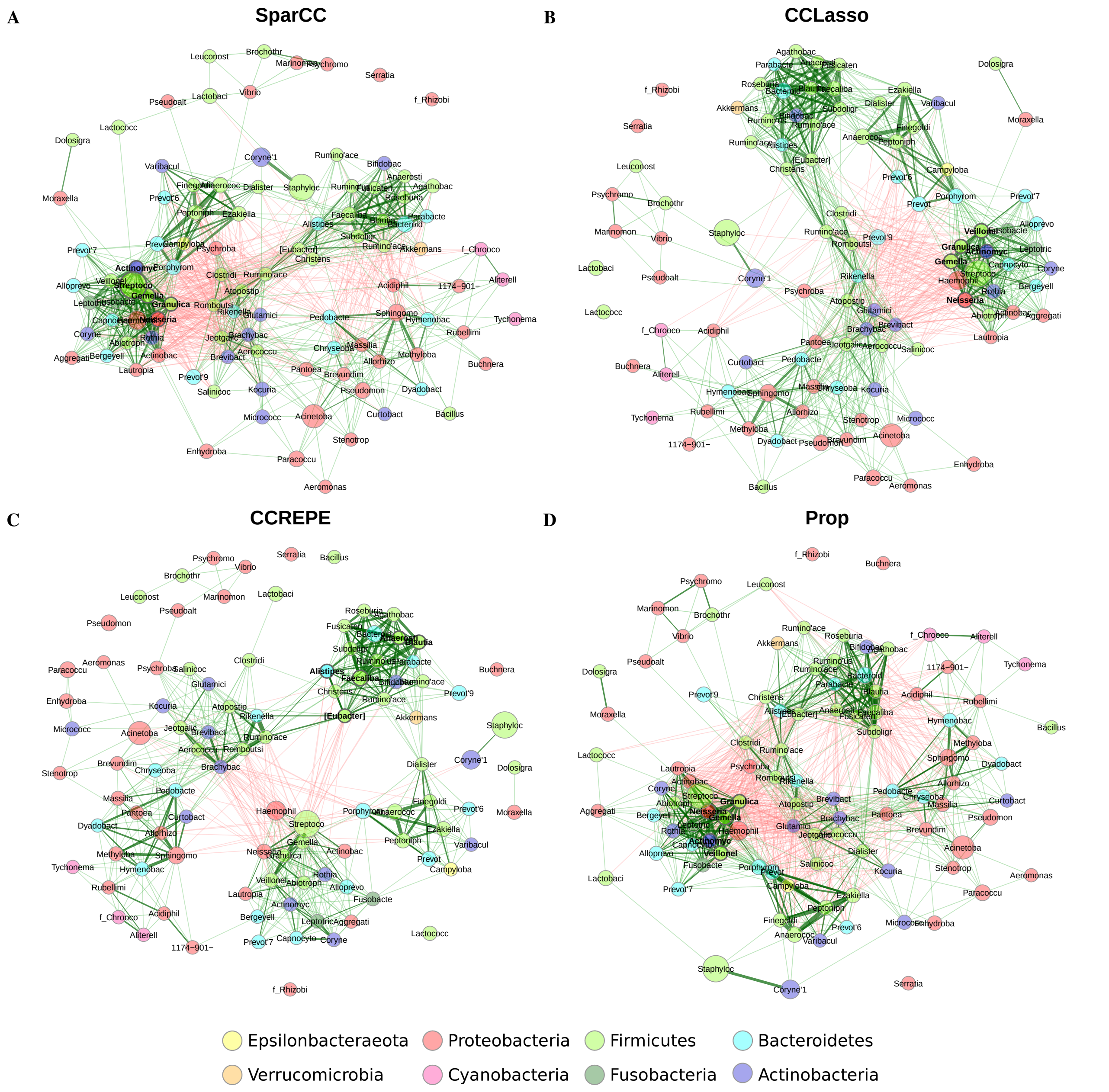

Figure S6: Microbial networks based on different association measures (three correlation measures and proportionality). For all four approaches, a threshold of $r = 0.2$ is used for network sparsification. For CCLasso, CCREPE, and Proportionality, a unit pseudo count is added to eliminate zero counts. The "signed" dissimilarity function is used to transform associations into dissimilarities. Green edges indicate positive correlations and red edges negative ones. Node color represents the phylum and node sizes the sum of relative frequencies of a genus. Hubs (highlighted by bold font and borders) are nodes with an eigenvector centrality above the empirical 95% quantile of all eigenvector centrality values.

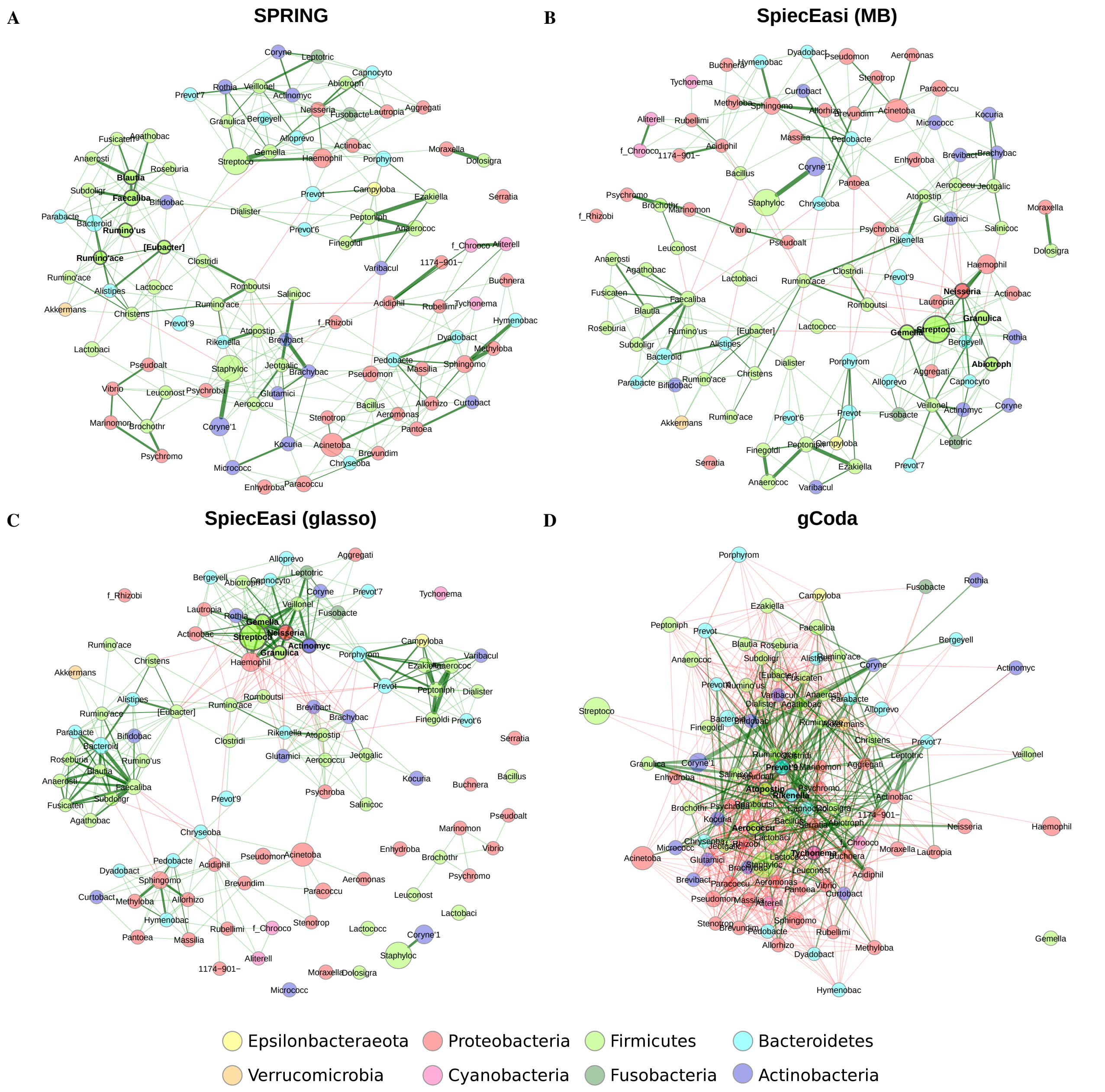

Figure S7: Microbial networks constructed using NetCoMi's conditional dependence measures for association estimation. Since all four methods include model selection approaches, no additional sparsification is needed. For gCoda, unit pseudo counts are added to the observed counts. SPRING and SpiecEasi include zero handling methods. Where possible, the arguments of the corresponding R functions are set to the same values to receive comparable results. The "signed" dissimilarity function is used to transform associations into dissimilarities. Green edges indicate positive correlations and red edges negative ones. Node color represents the phylum and node sizes the sum of relative frequencies of a genus. Hubs (highlighted by bold font and borders) are nodes with an eigenvector centrality above the empirical 95% quantile of all eigenvector centrality values.

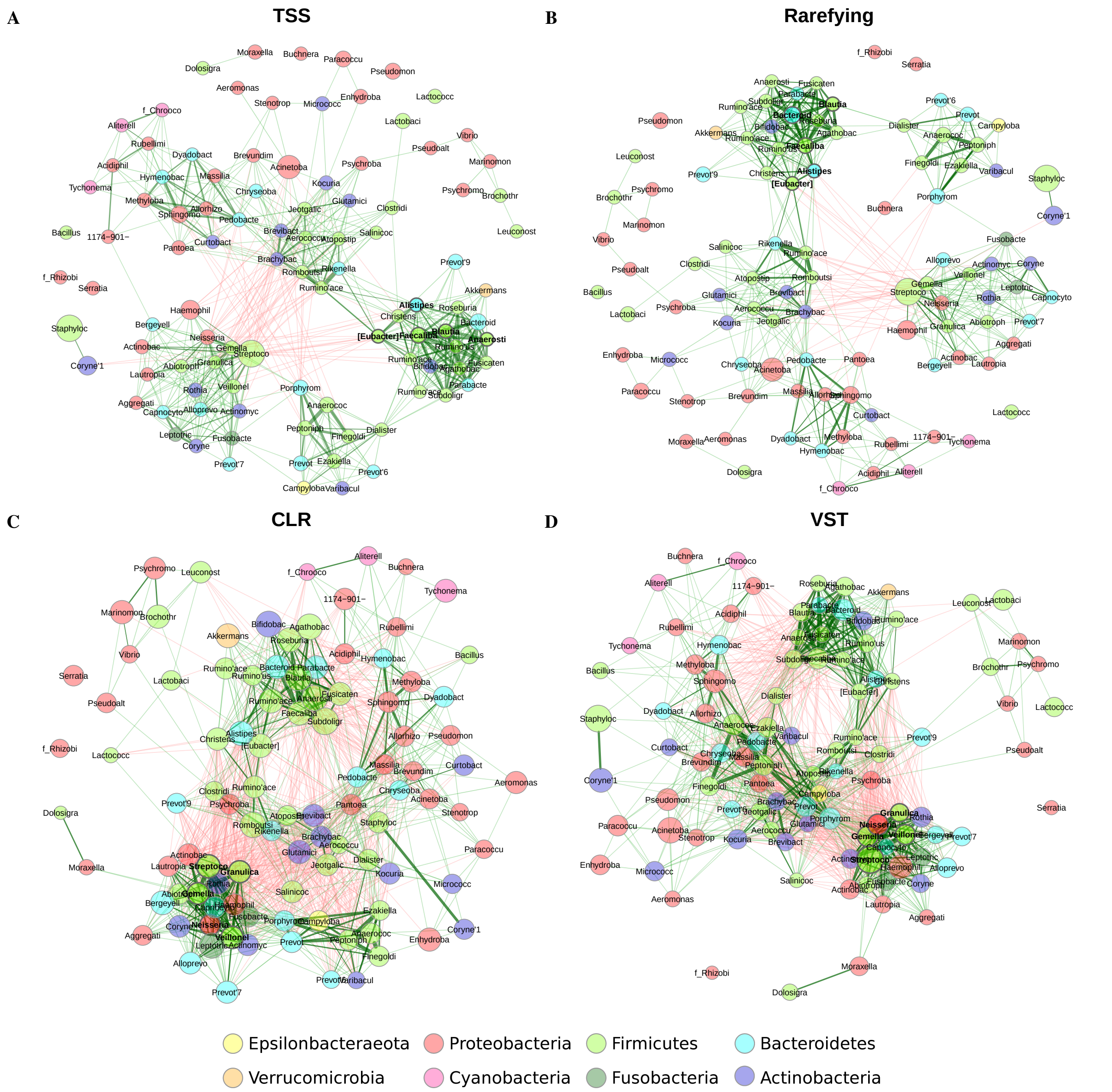

Figure S8: Microbial networks constructed using four different normalization methods available in NetCoMi. Pearson correlations are used as association measure. Even if TSS and rarefying can handle zeros in the data, a unit pseudo count is added to the observed counts in all four cases to improve comparability. A threshold of 0.2 is used for sparsification. The "signed" dissimilarity function is used to transform associations into dissimilarities. Green edges indicate positive correlations and red edges negative ones. Node color represents the phylum and node sizes the sum of relative frequencies of a genus. Hubs (highlighted by bold font and borders) are nodes with an eigenvector centrality above the empirical 95% quantile of all eigenvector centrality values.

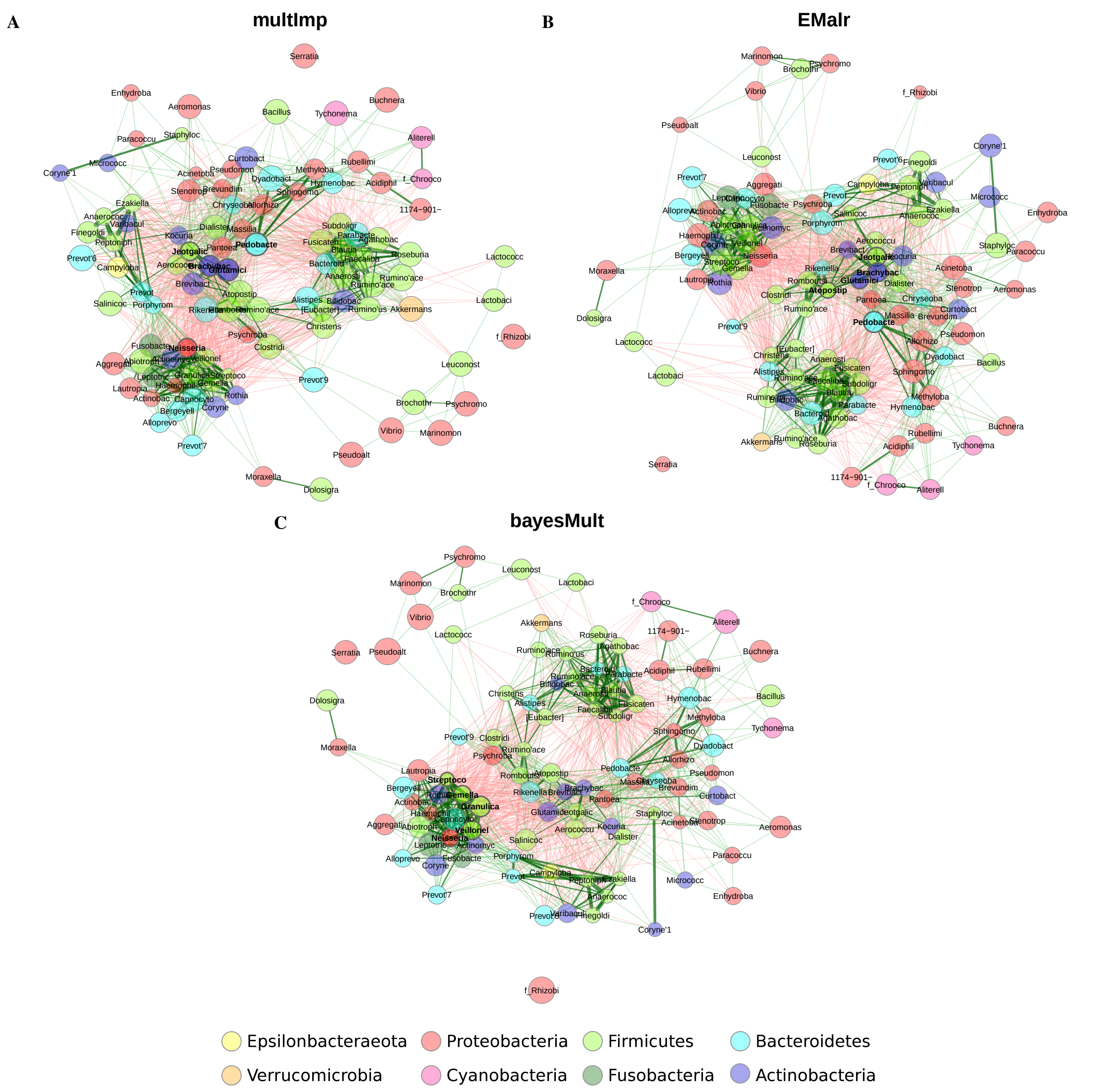

Figure S9: Microbial networks constructed using different zero handling methods available in NetCoMi. Pearson correlations are used as association measure and the observed counts are clr transformed after zero replacement. A threshold of 0.2 is used for sparsification. The "signed" dissimilarity function is used to transform associations into dissimilarities. Green edges indicate positive correlations and red edges negative ones. Node color represents the phylum and node sizes the sum of relative frequencies of a genus. Hubs (highlighted by bold font and borders) are nodes with an eigenvector centrality above the empirical 95% quantile of all eigenvector centrality values.

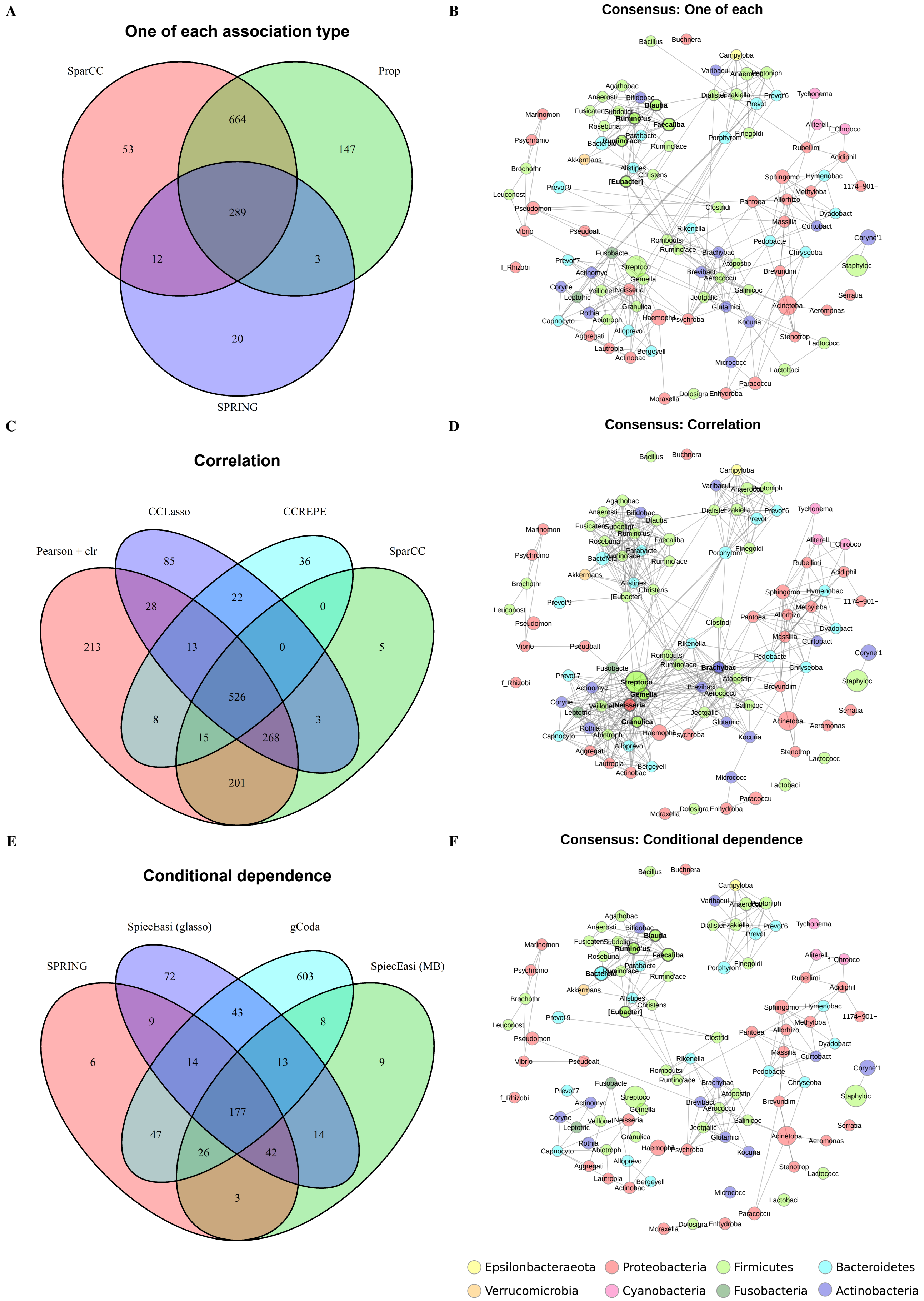

Figure S10: **A**, **C**, and **E**: Venn diagrams of overlapping edges within the three settings: "One of each association type", "Correlation measures", "Conditional dependence measures". **B**, **D**, and **F**: Consensus networks for the same three settings. An edge exists between nodes that are connected in all three or four networks of the respective setting. Node color represents the phylum and node sizes the sum of relative frequencies of a genus. Hubs (highlighted by bold font and borders) are nodes with an eigenvector centrality above the empirical 95% quantile of all eigenvector centrality values. All consensus networks use the layout computed for the "normalization" consensus network because it displays the best division into clusters.

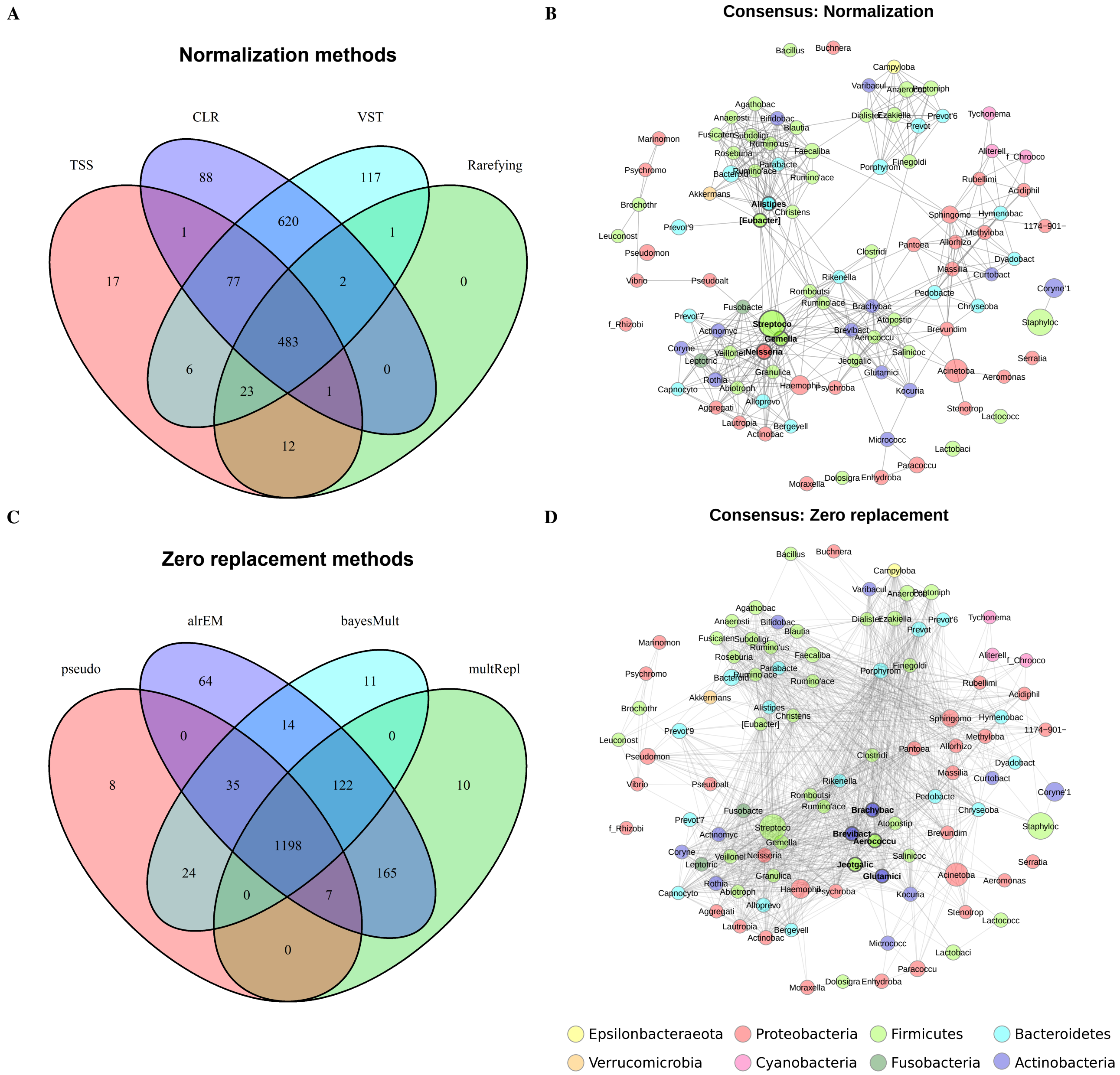

Figure S11: **A** and **C**: Venn diagrams of overlapping edges within the two settings: "Normalization methods" and "Zero replacement methods". **B** and **D**: Consensus networks for the same two settings. An edge exists between nodes that are connected in all four networks of the respective setting. Node color represents the phylum and node sizes the sum of relative frequencies of a genus. Hubs (highlighted by bold font and borders) are nodes with an eigenvector centrality above the empirical 95% quantile of all eigenvector centrality values. All consensus networks use the layout computed for the "normalization" consensus network because it displays the best division into clusters.

Table S15: Global network properties of the 15 networks considered in this section, divided into association types and preprocessing methods. Since single nodes are not removed from the networks, edge and vertex connectivity are zero for all networks and not shown in the table. In addition to NetCoMi's output, the total number of edges is given.

	Average path length	Clustering coefficient	Modularity	Edge density	Number of edges
**Correlation:**					
SparCC	1.323	0.623	0.264	0.206	1018
CCLasso	1.351	0.643	0.397	0.191	945
CCREPE	1.613	0.621	0.550	0.125	620
**Conditional dependence:**					
SPRING	2.386	0.384	0.655	0.065	324
SpiecEasi(MB)	2.179	0.360	0.640	0.059	292
SpiecEasi(glasso)	1.909	0.548	0.581	0.078	384
gCoda	1.381	0.361	0.242	0.188	931
**Proportionality:**					
Prop	1.306	0.628	0.283	0.223	1103
**Normalization methods:**					
TSS	1.613	0.621	0.550	0.125	620
Rarefying	1.822	0.656	0.613	0.105	522
CLR	1.225	0.632	0.272	0.257	1272
VST	1.214	0.641	0.233	0.268	1329
**Zero replacement methods:**					
multImp	1.166	0.643	0.196	0.303	1502
EMalr	1.121	0.642	0.220	0.324	1605
bayesMult	1.190	0.634	0.212	0.284	1404

Table S16: For each setting and each centrality value available in NetCoMi, the multiple-site similarity index (SimInd) proposed by (Diserud and Ødegaard, 2007) is shown. The index expresses the similarity of the sets of most central nodes within a setting. The set of most central nodes comprise nodes with a centrality value either above the 75% quantile or the 95% quantile of the empirical distribution of centralities. In addition, for each setting and centrality measure the intersection (Inters.) of all three or four sets of most central nodes is shown.

Setting	Degree		Betweenness centr.		Closeness centr.		Eigenvector centr.	
	SimInd	Inters.	SimInd	Inters.	SimInd	Inters.	SimInd	Inters.
**75% quantile:**								
Each of one	0.76	11	0.66	11	0.76	14	0.58	4
Correlation	0.86	13	0.75	9	0.84	16	0.69	5
Cond. dependence	0.66	3	0.60	3	0.59	5	0.53	3
Normalization	0.78	13	0.73	9	0.87	16	0.69	3
Zero replacement	0.92	18	0.78	11	0.92	20	0.92	19
**95% quantile:**								
Each of one	0.35	1	0.30	1	0.30	0	0.40	0
Correlation	0.59	1	0.53	2	0.53	0	0.60	0
Cond. dependence	0.30	0	0.47	1	0.40	0	0.27	0
Normalization	0.52	0	0.80	2	0.67	1	0.60	0
Zero replacement	0.74	2	0.80	3	0.60	1	0.67	0

8 Existing simulation studies comparing association and dissimilarity measures

Table S17: Exiting simulation studies, where the performance of the association measures considered in our work is evaluated and compared. Publications containing a simulation study, where one or more of the association measures used in our work is considered, are in rows and the association measures are in columns. The measures designed for sequencing data are ordered by publication date.

	Traditional measures				Developed for sequencing data						
	Pearson	Spearman	clr-Pearson	Bray-Curtis	CCREPE	SparCC	SPIEC-EASI	CCLasso	gCoda	proportionality	SPRING
Year of publication	-	-	-	-	2012	2012	2015	2015	2017	2017	2019
SparCC paper (Friedman and Alm, 2012)	x	x	x			x					
SPIEC-EASI paper (Kurtz et al., 2015)	x				x	x	x				
CCLasso paper (Fang et al., 2015)						x		x			
Review by Weiss et al. (Weiss et al., 2016)	x	x		x		x					
gCoda paper (Fang et al., 2017)							x		x		
propr paper (Quinn et al., 2017)	x		x						x		
Review by Röttjers and Faust (Röttjers and Faust, 2018)		x				x	x		x		
SPRING paper (Yoon et al., 2019)	x					x	x				x
Review by Hirano and Takemoto (Hirano and Takemoto, 2019)	x	x				x	x	x			

References

[Aitchison 1992] Aitchison, John: On Criteria for Measures of Compositional Difference. *Mathematical Geology* 24, Nr. 4: 365–379 (1992)

[Badri et al. 2020] Badri, Michelle ; Kurtz, Zachary D. ; Bonneau, Richard ; Müller, Christian L.: Shrinkage improves estimation of microbial associations under different normalization methods. *bioRxiv* (2020). `http://dx.doi.org/10.1101/406264`. – DOI 10.1101/406264

[Benjamini and Hochberg 2000] Benjamini, Yoav ; Hochberg, Yosef: On the adaptive control of the false discovery rate in multiple testing with independent statistics. *Journal of Educational and Behavioral Statistics* 25, Nr. 1: 60–83 (2000)

[Benjamini and Yekutieli 2001] Benjamini, Yoav ; Yekutieli, Daniel: The control of the false discovery rate in multiple testing under dependency. *The Annals of statistics* 29: 1165–1188 (2001)

[Bray and Curtis 1957] Bray, J R. ; Curtis, John T.: An ordination of the upland forest communities of southern Wisconsin. *Ecological monographs* 27, Nr. 4: 325–349 (1957)

[Dawson et al. 2012] Dawson, John A. ; Ye, Shuyun ; Kendziorski, Christina: R/EBcoexpress: an empirical Bayesian framework for discovering differential co-expression. *Bioinformatics* 28, Nr. 14: 1939–1940 (2012)

[Diserud and Ødegaard 2007] Diserud, Ola H. ; Ødegaard, Frode: A multiple-site similarity measure. *Biology Letters* 3, Nr. 1: 20–22 (2007)

[Efron 2005] Efron, Bradley: Local False Discovery Rates. Version: 2005. `http://statweb.stanford.edu/ ckirby/brad/papers/2005LocalFDR.pdf`. 2005. – Forschungsbericht

[Endres and Schindelin 2003] Endres, Dominik M. ; Schindelin, Johannes E.: A New Metric for Probability Distributions. *IEEE Transactions on Information Theory* 49, Nr. 7 (2003)

[Fang 2016] Fang, Huaying: *CCLasso: Correlation Inference for Compositional Data through Lasso*. `https://github.com/huayingfang/CCLasso`, 2016

[Fang et al. 2015] Fang, Huaying ; Huang, Chengcheng ; Zhao, Hongyu ; Deng, Minghua: CCLasso: correlation inference for compositional data through Lasso. *Bioinformatics* 31, Nr. 19: 3172–3180 (2015)

[Fang et al. 2017] Fang, Huaying ; Huang, Chengcheng ; Zhao, Hongyu ; Deng, Minghua: gCoda: Conditional Dependence Network Inference for Compositional Data. *Journal of Computational Biology* 24, Nr. 7: 699–708 (2017)

[Faust et al. 2012] Faust, Karoline ; Sathirapongsasuti, J F. ; Izard, Jacques ; Segata, Nicola ; Gevers, Dirk ; Raes, Jeroen ; Huttenhower, Curtis: Microbial Co-occurrence Relationships in the Human Microbiome. *PLoS Computational Biology* 8, Nr. 7: e1002606 (2012)

[Filosi 2017] Filosi, Michele: *r-sparcc*. `https://github.com/MPBA/r-sparcc`, 2017

[Fisher 1915] Fisher, Ronald A.: Frequency distribution of the values of the correlation coefficient in samples from an indefinitely large population. *Biometrika* 10: 507–521 (1915)

[Fisher 1921] Fisher, Ronald A.: On the "probable error" of a coefficient of correlation deduced from a small sample. *Metron* 1: 3–32 (1921)

[Fisher 1992] Fisher, Ronald A.: Statistical methods for research workers. *Breakthroughs in Statistics*. Springer, 1992, 66–70

[Friedman and Alm 2012] Friedman, Jonathan ; Alm, Eric J.: Inferring Correlation Networks from Genomic Survey Data. *PLoS Computational Biology* 8: e1002687 (2012)

[Gill et al. 2010] Gill, Ryan ; Datta, Somnath ; Datta, Susmita: A statistical framework for differential network analysis from microarray data. *BMC Bioinformatics* 11: 95 (2010)

[Gill et al. 2014] Gill, Ryan ; Datta, Somnath ; Datta, Susmita: Differential Network Analysis in Human Cancer Research. *Current Pharmaceutical Design* 20: 4–10 (2014)

[Han et al. 2011] Han, Jiawei ; Pei, Jian ; Kamber, Micheline: *Data mining: concepts and techniques*. Elsevier, 2011

[Hirano and Takemoto 2019] Hirano, Hokuto ; Takemoto, Kazuhiro: Difficulty in inferring microbial community structure based on co-occurrence network approaches. *BMC bioinformatics* 20, Nr. 1: 329 (2019)

[Hosseini et al. 2012] Hosseini, SM H. ; Hoeft, Fumiko ; Kesler, Shelli R.: GAT: A Graph-Theoretical Analysis Toolbox for Analyzing Between-Group Differences in Large-Scale Structural and Functional Brain Networks. *PloS One* 7: e40709 (2012)

[Jaccard 1908] Jaccard, Paul: Nouvelles Recherches Sur la Distribution Florale. *Bulletin de la Société Vaudoise des Sciences Naturelles* 44: 223–270 (1908)

[Jeffreys 1948] Jeffreys, Harold: Theory of probability. (1948)

[Johnson and Sinanovic 2001] Johnson, Don ; Sinanovic, Sinan: Symmetrizing the Kullback-Leibler Distance. *IEEE Transactions on Information Theory* (2001)

[Klaus and Strimmer. 2015] Klaus, Bernd ; Strimmer., Korbinian: *fdrtool: Estimation of (Local) False Discovery Rates and Higher Criticism*, 2015. `https://CRAN.R-project.org/package=fdrtool`. – R package version 1.2.15

[Kullback and Leibler 1951] Kullback, Solomon ; Leibler, Richard A.: On information and sufficiency. *The Annals of Mathematical Statistics* 22, Nr. 1: 79–86 (1951)

[Kurtz et al. 2015] Kurtz, Zachary D. ; Müller, Christian L. ; Miraldi, Emily R. ; Littman, Dan R. ; Blaser, Martin J. ; Bonneau, Richard A.: Sparse and Compositionally Robust Inference of Microbial Ecological Networks. *PLoS Computational Biology* 11: e1004226 (2015)

[Langaas et al. 2005] Langaas, Mette ; Lindqvist, Bo H. ; Ferkingstad, Egil: Estimating the proportion of true null hypotheses, with application to DNA microarray data. *Journal of the Royal Statistical Society: Series B (Statistical Methodology)* 67, Nr. 4: 555–572 (2005)

[Langfelder and Horvath 2008] Langfelder, Peter ; Horvath, Steve: WGCNA: an R package for weighted correlation network analysis. *BMC Bioinformatics* 9, Nr. 1: 559 (2008)

[Langfelder et al. 2016] Langfelder, Peter ; Zhang, Bin ; Steve Horvath with contributions f.: *dynamicTreeCut: Methods for Detection of Clusters in Hierarchical Clustering Dendrograms*, 2016. `https://CRAN.R-project.org/package=dynamicTreeCut`. – R package version 1.63-1

[Liu et al. 2012] Liu, Han ; Han, Fang ; Yuan, Ming ; Lafferty, John ; Wasserman, Larry et al.: High-dimensional semiparametric Gaussian copula graphical models. *The Annals of Statistics* 40, Nr. 4: 2293–2326 (2012)

[Marchette 2005] Marchette, David J.: *Random graphs for statistical pattern recognition*. Bd. 565. John Wiley & Sons, 2005

[Marchette 2015] Marchette, David J.: *cccd: Class Cover Catch Digraphs*, 2015. `https://CRAN.R-project.org/package=cccd`. – R package version 1.5

[Marozzi 2004] Marozzi, Marco: Some remarks about the number of permutations one should consider to perform a permutation test. *Statistica* 64: 193–201 (2004)

[Martín-Fernández et al. 1999] Martín-Fernández, Josep A. ; Bren, Mark J. ; Barceló-Vidal, Carles ; Pawlowsky-Glahn, Vera: A measure of difference for compositional data based on measures of divergence. *Lippard, Næss, and Sinding-Larsen* (1999)

[Mason et al. 2009] Mason, Mike J. ; Fan, Guoping ; Plath, Kathrin ; Zhou, Qing ; Horvath, Steve: Signed weighted gene co-expression network analysis of transcriptional regulation in murine embryonic stem cells. *BMC Genomics* 10, Nr. 1: 327 (2009)

[McKenzie et al. 2016] McKenzie, Andrew T. ; Katsyv, Igor ; Song, Won-Min ; Wang, Minghui ; Zhang, Bin: DGCA: A comprehensive R package for Differential Gene Correlation Analysis. *BMC Systems Biology* 10: 106 (2016)

[Pesarin 2001] Pesarin, Fortunato: *Multivariate Permutation Tests: With Applications to Biostatistics*. John Wiley & Sons, New York, 2001

[Phipson and Smyth 2010] Phipson, Belinda ; Smyth, Gordon K.: Permutation P-values should never be zero: calculating exact P-values when permutations are randomly drawn. *Statistical Applications in Genetics and Molecular Biology* 9, Nr. 1 (2010)

[Qannari et al. 2014] Qannari, El M. ; Courcoux, Philippe ; Faye, Pauline: Significance test of the adjusted Rand index. Application to the free sorting task. *Food Quality and Preference* 32: 93–97 (2014)

[Quinn et al. 2017] Quinn, Thomas P. ; Richardson, Mark F. ; Lovell, David ; Crowley, Tamsyn M.: propr: an R-package for Identifying Proportionally Abundant Features Using Compositional Data Analysis. *Scientific reports* 7, Nr. 1: 16252 (2017)

[Rand 1971] Rand, William M.: Objective criteria for the evaluation of clustering methods. *Journal of the American Statistical Association* 66, Nr. 336: 846–850 (1971)

[Real 1999] Real, Raimundo: Tables of significant values of Jaccard's index of similarity. *Miscellania Zoologica* 22: 29–40 (1999)

[Real and Vargas 1996] Real, Raimundo ; Vargas, Juan M.: The Probabilistic Basis of Jaccard's Index of Similarity. *Systematic Biology* 45: 380–385 (1996)

[Röttjers and Faust 2018] Röttjers, Lisa ; Faust, Karoline: From hairballs to hypotheses–biological insights from microbial networks. *FEMS Microbiology Reviews* 42, Nr. 6: 761–780 (2018)

[Schwager et al. 2017] Schwager, Emma ; Mallick, Himel ; Ventz, Steffen ; Huttenhower, Curtis: A Bayesian method for detecting pairwise associations in compositional data. *PLoS Comput Biol* 13, Nr. 11: e1005852 (2017)

[Siska et al. 2016] Siska, Charlotte ; Bowler, Russell ; Kechris, Katerina: The discordant method: a novel approach for differential correlation. *Bioinformatics* 32: 690–696 (2016)

[Siska and Kechris 2019] Siska, Charlotte ; Kechris, Katerina: *discordant: The Discordant Method: A Novel Approach for Differential Correlation*, 2019. `https://github.com/siskac/discordant`. – R package version 1.6.1

[Uehara et al. 2014] Uehara, Taira ; Yamasaki, Takao ; Okamoto, Tsuyoshi ; Koike, Takahiko ; Kan, Shigeyuki ; Miyauchi, Satoru ; Kira, Jun-ichi ; Tobimatsu, Shozo: Efficiency of a "Small-World" Brain Network Depends on Consciousness Level: A Resting-State fMRI Study. *Cerebral Cortex* 24: 1529–1539 (2014)

[Warnes et al. 2018] Warnes, Gregory R. ; Bolker, Ben ; Lumley, Thomas: *gtools: Various R Programming Tools*, 2018. `https://CRAN.R-project.org/package=gtools`. – R package version 3.8.1

[Weiss et al. 2016] Weiss, Sophie ; Van Treuren, Will ; Lozupone, Catherine ; Faust, Karoline ; Friedman, Jonathan ; Deng, Ye ; Xia, Li C. ; Xu, Zhenjiang Z. ; Ursell, Luke ; Alm, Eric J. et al.: Correlation detection strategies in microbial data sets vary widely in sensitivity and precision. *The ISME Journal* 10, Nr. 7: 1669–1681 (2016)

[Yoon et al. 2018] Yoon, Grace ; Carroll, Raymond J. ; Gaynanova, Irina: *Sparse semiparametric canonical correlation analysis for data of mixed types*. 2018

[Yoon et al. 2019] Yoon, Grace ; Gaynanova, Irina ; Müller, Christian L.: Microbial networks in SPRING-Semi-parametric rank-based correlation and partial correlation estimation for quantitative microbiome data. *Frontiers in Genetics* 10 (2019)

[Zhang and Horvath 2005] Zhang, Bin ; Horvath, Steve: A General Framework for Weighted Gene Co-Expression Network Analysis. *Statistical Applications in Genetics and Molecular Biology* 4: 17 (2005)
